# Discovery of Chalcone-Based Hybrid Structures as High Affinity and Site-Specific Inhibitors against SARS-CoV-2: A Comprehensive Structural Analysis Based on Various Host-Based and Viral Targets

**DOI:** 10.3390/ijms24108789

**Published:** 2023-05-15

**Authors:** Mehdi Valipour, Silvia Di Giacomo, Antonella Di Sotto, Hamid Irannejad

**Affiliations:** 1 Razi Drug Research Center, Iran University of Medical Sciences, Tehran 1545913487, Iran; 2 Department of Physiology and Pharmacology “V. Erspamer”, Sapienza University of Rome, P.le Aldo Moro 5, 00185 Rome, Italy; 3 Department of Medicinal Chemistry, Faculty of Pharmacy, Mazandaran University of Medical Sciences, Sari 4847116547, Iran

**Keywords:** chalcone, SARS-CoV-2, 3CLpro, PLpro, site-specific inhibitor, host-based targets, MM/PB(GB)SA

## Abstract

Previous studies indicated that natural-based chalcones have significant inhibitory effects on the coronavirus enzymes 3CLpro and PLpro as well as modulation of some host-based antiviral targets (HBATs). In this study, a comprehensive computational and structural study was performed to investigate the affinity of our compound library consisting of 757 chalcone-based structures (CHA-1 to CHA-757) for inhibiting the 3CLpro and PLpro enzymes and against twelve selected host-based targets. Our results indicated that CHA-12 (VUF 4819) is the most potent and multi-target inhibitor in our chemical library over all viral and host-based targets. Correspondingly, CHA-384 and its congeners containing ureide moieties were found to be potent and selective 3CLpro inhibitors, and benzotriazole moiety in CHA-37 was found to be a main fragment for inhibiting the 3CLpro and PLpro. Surprisingly, our results indicate that the ureide and sulfonamide moieties are integral fragments for the optimum 3CLpro inhibition while occupying the S1 and S3 subsites, which is fully consistent with recent reports on the site-specific 3CLpro inhibitors. Finding the multi-target inhibitor CHA-12, previously reported as an LTD4 antagonist for the treatment of inflammatory pulmonary diseases, prompted us to suggest it as a concomitant agent for relieving respiratory symptoms and suppressing COVID-19 infection.

## 1. Introduction

The Coronavirus disease 2019 (COVID-19) is one of the most terrible viral diseases that mankind has ever faced.

Treatment of COVID-19 requires careful consideration of cellular and molecular mechanisms to accurately identify the components as well as pathogenic mechanisms of the SARS-CoV-2 [1]. Structural and functional studies have shown that the virus is composed of various structural and non-structural proteins (NSPs) [2]. The virus has four major glycoproteins in its structure: spike protein (S), membrane protein (M), envelope protein (E), and nucleocapsid (N) protein. SARS-CoV-2 spike (S) glycoproteins attach to appropriate host cells and cause virus entry, while proteins M, E, and N are more involved in the assembly, replication, release of the virus particles, and physical protection [3]. In addition to structural proteins, 15 NSPs have been identified for the SARS-CoV-2 so far [4]. Depending on the importance of their role in the survival and replication of the virus, each of them can be considered as a potential target. The 3-chymotrypsin-like protease (3CLpro) and the papain-like protease (PLpro) enzymes are the most important NSPs that play a vital role in the processing of SARS-CoV-2 polyproteins and the production of the other NSPs [5]. These proteases are being considered by scientists as important targets for the design of SARS-CoV-2 inhibitors [6,7,8], and the appropriate selection of these targets for the treatment of COVID-19 is strongly confirmed in the results of new clinical trials [9].

Recently, the active site of the SARS-CoV-2 3CLpro was well characterized, and it was realized that the active site has four subsites named S1, S1’, S2, and S3. The 3CLpro is a cysteine protease, and His41 and Cys145 comprise the catalytic dyad. Dimerization of the enzyme completes the active site and forms the oxyanion hole for substrate specificity. The S1 subsite has two key residues His163, which is interacting with the pyridine nitrogen or with nitrogen-containing heterocycles, and Glu166, which is a key amino acid in the formation of hydrogen bonds to the amide or ureide substructures of the inhibitors. The S2 subsite of SARS-CoV-2 has a small and hydrophobic space termed the “aromatic wheel” consisting mainly of Met49 and Met165. The S3 subsite has been revealed to be explored and occupied by the sulfonamide group of the inhibitors and interacts with hydrogen bonding to Gln189. This space is exposed to the solvent, and fragments located in this space can be oriented to the solvent. This space consists of His164, Met165, and Asp187. These subsites are mainly used for the design of novel inhibitors simultaneously occupying two or three subsites for higher affinity by fragment merging or growing [10]. By evaluating the active site of SARS-CoV-2 3CLpro, some recent studies have also re-viewed the structural basis of its potential inhibitors, which is useful for the targeted design of anti-COVID-19 therapeutic agents in future works [11].

Although many direct-acting antiviral drugs (DAADs) are designed to inhibit virus components, overuse of these agents can eventually lead to undesirable effects such as drug resistance [12,13]. Viruses broadly use host cell capabilities known as host-based antiviral targets (HBATs) to replicate their own particles. Therefore, targeted regulation of these features can help to inhibit virus pathogenicity. Because HBATs are not genetically controlled by the virus genome, it is expected that inhibiting viruses through regulating these targets does not lead to drug resistance [14].

Almost, three years after the emergence of SARS-CoV-2, the management of HBATs for the treatment of COVID-19 has become of great interest to scientists [15]. Due to the homology and high genetic similarity of SARS-CoV-2 to SARS-CoV (~80% similarity) and MERS-CoV (~50% similarity), it is well established that these viruses use a similar mechanism to enter the host cells and infect their host by binding to the angiotensin-converting enzyme II (ACE2) receptors on the surface of the host cell [16,17,18,19,20]. Disrupting host cell entry, which is the first step in the viral life cycle, can be a sensible treatment and preventive strategy [21]. The role of the host cathepsin cysteine protease enzyme is crucial for infecting ACE2 receptor-containing cells and the entry of the SARS-CoV-2 through endocytosis. Therefore, cathepsin L could be considered as a high-potential HBAT for therapeutic interventions [22]. The p38 Mitogen-Activated Protein Kinase (p38 MAPK) is another highlighted HBAT for the treatment of COVID-19. Since this signaling pathway plays a critical role in host-inflammatory responses and release of cytokines and is also regulated by coronaviruses for infection, accordingly, this pathway was introduced by scientists as a promising target for the treatment of COVID-19 [23]. Cyclin-dependent kinases (CDK) are another highlighted HBAT for the treatment of COVID-19 and play a prominent role in cell proliferation. Basically, viruses alter the functioning of CDKs to favor viral replication and, hence, some recent studies considered the development of CDK inhibitors to be an important strategy for achieving broad-spectrum antivirals that can be used to treat COVID-19 [24]. Some scientists have recently proposed dihydroorotate dehydrogenase (DHODH) as an attractive HBAT for the development of broad-spectrum antivirals, which provides more treatment options for COVID-19 through its dual mechanism of antiviral action and immune system regulation [25].

So far, scientists have introduced various structures and scaffolds for the design and development of anti-SARS-CoV-2 agents [26,27]. Computational and in silico methods have also been used to find suitable anti-COVID-19 agents from nature [28,29,30,31,32]. Some of these studies have focused on identifying the capabilities of chalcone structures (or their rigid structural analogues, i.e., flavonoids) to combat SARS-CoV-2 [33]. In continuation of our efforts to find effective treatments against COVID-19 [34,35,36,37], we recently introduced the chalcone-amide scaffold as an excellent backbone for the design and development of selective SARS-CoV/SARS-CoV-2 PLpro inhibitors and even explained that this scaffold can also be used for designing the SARS-CoV-2 3CLpro inhibitors [38].

**Figure 1:**
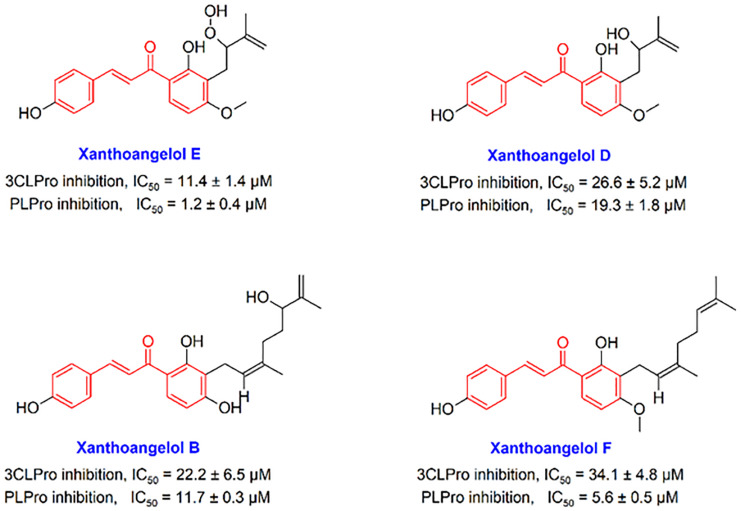
Chemical structures of some natural-based chalcones having anti-SARS-CoV activity.

In the present study, we focus on the chalcone scaffold, which is one of the most important scaffolds used in medicinal chemistry and is abundant in the chemical structure of many compounds of natural origin, to which it confers a number of bioactivities, among which are antioxidant, anti-inflammatory, and antiviral ones [39]. In recent years, chalcones have attracted great attention as possible novel antiviral agents, due to their broad-spectrum activity and low toxicity potential [40]. For instance, xanthohumol, a prenylated chalcone isolated from *Humulus lupulus* L., has been shown to possess interesting antiviral properties and to act as a potent pan-inhibitor of coronaviruses; moreover, anti-coronavirus properties have been reported for chalcones isolated from *Glycyrrhiza* spp. and from *Angelica keiskei* (Miq.) Koidz. [39,41,42]. Structurally, chalcone is an analogue of the chalcone-amide, which contains an α, β-unsaturated ketone functional group, which allows this scaffold to participate in Michael’s reaction and create strong covalent bonds at the active sites of the viral cysteine proteases. Although there are some studies indicating that chalcone-based compounds have a significant ability to inhibit coronaviruses enzymes 3CLpro and PLpro, the recruitment of this interesting scaffold has been neglected in the discovery and development of anti-SARS-CoV-2 agents. In previous investigations, some chalcones have been introduced that showed significant potential to inhibit SARS-CoV 3CLpro and PLpro enzymes. In a study by Park et al., it was found that some natural-based chalcones Xanthoangelol E, Xanthoangelol D, Xanthoangelol B, and Xanthoangelol F (Figure 1) exert anti-SARS-CoV activity by inhibiting the 3CLpro and PLpro enzymes through competitive (for 3CLpro) and non-competitive (for PLpro) inhibitory mechanisms [41]. Some other studies have also reported that chalcone-based structures have important effects in inhibiting these enzymes [43,44,45]. In addition, some studies have reported that chalcones can disrupt vital processes such as virus replication by modulating HBATs [40]. Recently, the capabilities and weaknesses of chalcones in the drug discovery and development of anti-SARS-CoV-2 agents have been reviewed in detail [46]. In the study, it has been proposed that by placing the α, β-unsaturated moiety of chalcone in the appropriate position of optimized anti-SARS-CoV-2 agents, it may be possible to irreversibly inhibit 3CLpro and PLpro cysteine proteases through Michael reaction. Given that many synthetic or natural derivatives of chalcones have been identified so far, it makes sense to search for finding new and active derivatives of chalcone-based structures against SARS-CoV-2.

In the current study, we have tried to find and identify active chalcones against the SARS-CoV-2 PLpro and 3CLpro enzymes within a compound library of 757 chalcone-based structures using computational methods, and we also tried to evaluate structural relationships between these compounds and previously reported inhibitors. In addition, the affinity of these compounds against a wide range of HBATs was evaluated in silico to identify hit compounds with the highest potential therapeutic activity. Subsequently, a part of the aim of this study was to discover and propose subsite-specific inhibitors of the 3CLpro and PLpro targets.

In the current study, a wide range of important HBATs (12 targets) that were previously reported as important targets for the treatment of viral diseases including COVID-19 were selected. These targets are cathepsin L, various types of CDKs, p38 MAPK, histone deacetylase 2 (HDAC2), dihydroorotate dehydrogenase (DHODH), bromodomain-containing proteins (BRDs), and sigma-1 receptor. Table S1 lists the specific HBATs and their PDB code considered in this study along with the chemical structure of their co-crystal ligands; it also cites credible articles that highlight the importance of these targets as key HBATs for the treatment of viral diseases such as COVID-19.

Accordingly, chalcone-based structures were screened virtually by molecular docking over the 14 molecular targets reported in Table S1, and the best hits were ranked and selected based on their docking score. The novelty of our strategy is due to the fact that we looked for chalcones that could simultaneously act against both virus- and host-based targets because the treatment of COVID-19 without considering host-based targets has led to failure. Ranking and selection were performed based on criteria described later in this study, and the best hits were selected for molecular dynamics and then Molecular Mechanics/Poisson Boltzmann (Generalized Born) Surface Area (MM/PB(GB)SA) analysis. Finally, a literature study has been done for the introduced ultimate leads, and their experimentally reported biological activities are discussed.

## 2. Results

### 2.1. Virtual Screening

**Figure 2:**
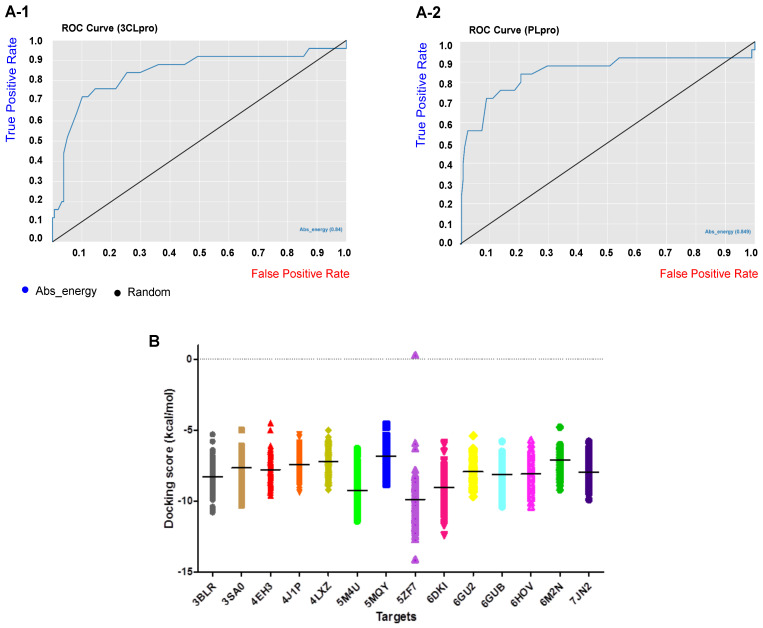
The ROC curve (**A-1**) 3CLpro and (**A-2**) PLpro benchmarking datasets. The area under the curve is 0.84 and 0.849 for 3CLpro and PLpro, respectively. (**B**) Docking score (kcal/mol) distribution of the 757 chalcone-based compound library over the 12 host-based molecular targets consisted of CDK9/cyclinT1 (3BLR), ERK2 (3SA0), p38 MAPK (4EH3), RBD2 (4J1P), HDAC2 (4LXZ), CK2 alpha’ (5M4U), Cathepsin L (5MQY), DHODH (5ZF7), Sigma-1 receptor (6DK1), CDK1 (6GU2), CDK2/CyclinA (6GUB), and RBD4 (6HOV) and the two viral targets 3CLpro (6M2N) and PLpro (7JN2). The intersecting line in each cluster represents the average docking score in each target.

Benchmarking datasets are very crucial to evaluate the performance and reliability of the virtual screening method. Benchmarking datasets are composed of known active compounds together with assumed non-active compounds referred to as “decoys”. The selection of decoys in benchmarking datasets was performed by the Knime Analytics Platform as described in the methods section. Accordingly, 25 active compounds against 3CLpro and PLpro were collected from the literature and used in the Knime workflow to search a ZINC database of 7000 dissimilar compounds to find decoys that are similar to the active compounds by the molecular descriptors MW, nHBDs, nHBAs, logP, and nRTB but are topologically different in 2D and 3D fingerprints. The Tanimoto index was measured as a fingerprint similarity to find the most dissimilar compounds and, accordingly, 158 and 189 decoys were selected for 3CLpro and PLpro targets, respectively, and were used for benchmarking our structured-based virtual screening method. In total, 183 compounds for 3CLpro and 214 compounds for PLpro were used as a benchmarking dataset. The ROC curves as a common metric for evaluating the performance of our virtual screening method are represented in Figure 2(A-1,A-2) for 3CLpro and PLpro targets. As seen, the area under the curve for the 3CLpro and PLpro benchmarking datasets are 0.84 and 0.849, respectively, and indicates the robustness of our virtual screening method to retrieve true positive (active) compounds. Overall accuracy statistics are also represented in Table 1 for the 3CLpro and PLpro benchmarking datasets.

Chalcone-based structures were searched in the PubChem database and after applying Lipinski’s rule of five, 757 chalcone derivatives were downloaded and used for virtual screening by AutoDock Vina over the 14 selected host and virus-based targets represented in Table S1 (see Supplementary File). The targets used are p38 MAPK, Cathepsin L, CDK1, CDK2/CyclinA, CDK9/cyclinT1, ERK2, HDAC2, DHODH, CK2 alpha, BRD2, BRD4, and sigma-1 receptor and virus-based targets 3CLpro and PLpro. Standard inhibitors baicalein (a selective 3CLpro inhibitor and the co-crystal ligand of PDB code: 6M2N), GRL0617 (a selective PLpro inhibitor), and hirsutenone (a dual inhibitor of the 3CLpro and PLpro) were used in our computational protocol as reference compounds, and validation was performed through re-dock of the co-crystal ligands in each target.

Docking scores of our compound library over the host and virus-based molecular targets are represented in Figure 2B, while each compound is presented by a single sign and each target by a different color. As shown in the figure, on average, the chalcone-based compounds showed higher affinities (lower docking score) towards the active site of the targets DHODH (5ZF7), CK2 alpha’ (5M4U), sigma-1 receptor (6DKI), and CDK9/cyclinT1 (3BLR), respectively, compared to other HBATs. Although, the average docking scores obtained for 3CLpro and PLpro enzymes are not remarkable; this is probably caused by the presence of a superficial active site of these enzymes, which is not a deep cavity. Comparison of the identified most active chalcones towards the two viral targets with their well-known standard inhibitors shows that these chalcones have a significant potential for the inhibition of the SARS-CoV-2 3CLpro and PLpro enzymes. Structural evaluation and analysis of these compounds led us to interesting results, which will be discussed in the following.

In order to choose the best hits with the lowest docking score (higher affinity), two selection criteria were applied. First, the structures that were ranked 1 to 5 in their docking score with the active site of each HBATs were identified (Table 2). Second, those compounds which were ranked 1 to 10 in their docking score with the active site of the 3CLpro and PLpro, were also identified and selected. The wider range of choosing compounds (rank 1 to 10) was applied for the 3CLpro and PLpro in order to focus more precisely on the viral targets, and hence structures that had a better rank in the 3CLpro and PLpro active sites were considered separately, regardless of their ranking in the host-based targets (Table 2). Finally, compounds that generated higher rankings with both host and virus-based targets were selected and highlighted for further structural and computational evaluations. For example, CHA-37 (rank 3 in PLpro with PDB code of 7JN2) was prioritized over CHA-282 (rank 2 in PLpro with PDB code of 7JN2) due to the higher ranking of CHA-37 over the host-based targets compared to CHA-282.

**Figure 3:**
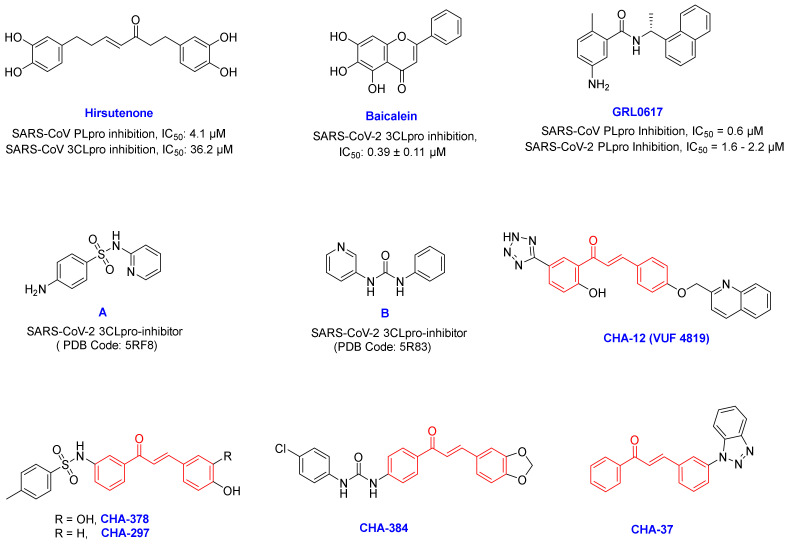
Chemical structure of some of the famous SARS-CoV-2 inhibitors hirsutenone, baicalein, and GRL0617 and their experimentally reported potencies, and also newly reported SARS-CoV-2 3CLpro fragment inhibitors A and B. The most active compounds CHA-12, CHA-37, CHA-297, CHA-378, and CHA-384 were selected as the promising chalcone-based structures for the inhibition of SARS-CoV-2 and identified by our preliminary in silico screening protocol.

By filtering the compounds through the selection criteria, some structures were identified that could selectively create remarkable interactions in the active sites of the 3CLpro or PLpro targets. While some compounds were found to simultaneously create strong interactions on both the SARS-CoV-2 targets. Among all 757 chalcone-based compounds in the library, some compounds such as CHA-15, CHA-236, CHA-383, CHA-384, CHA-392, CHA-397, and CHA-485 showed selective affinity in the 3CLpro active site. On the other hand, CHA-7, CHA-11, CHA-37, CHA-44, and CHA-177 showed selectivity to the PLpro active site, and CHA-12, CHA-37, CHA-297, and CHA-378 showed interesting potential for simultaneous inhibition of both the 3CLpro and PLpro targets. In our opinion, having the high potential for simultaneous inhibition of the HBATs along with the viral enzymes is an important criterion for the selection of ligands for subsequent evaluations. For example, CHA-86, CHA-118, CHA-233, and CHA-282, were not selected for further study due to their lack of potential to inhibit HBATs despite their high potential for inhibiting 3CLpro and PLpro enzymes. Among all compounds in our library, CHA-12, CHA-37, CHA-297, CHA-378, and CHA-384 are interesting compounds that generated an excellent binding profile in the active sites of most targets (Figure 3). Structural analysis and binding free energy calculations of these ligands in the viral targets showed interesting structure –activity relationships that are consistent with the experimental results previously reported for SARS-CoV/SARS-CoV-2 inhibitors.

### 2.2. Molecular Dynamics and MM/PB(GB)SA Analysis

**Figure 4:**
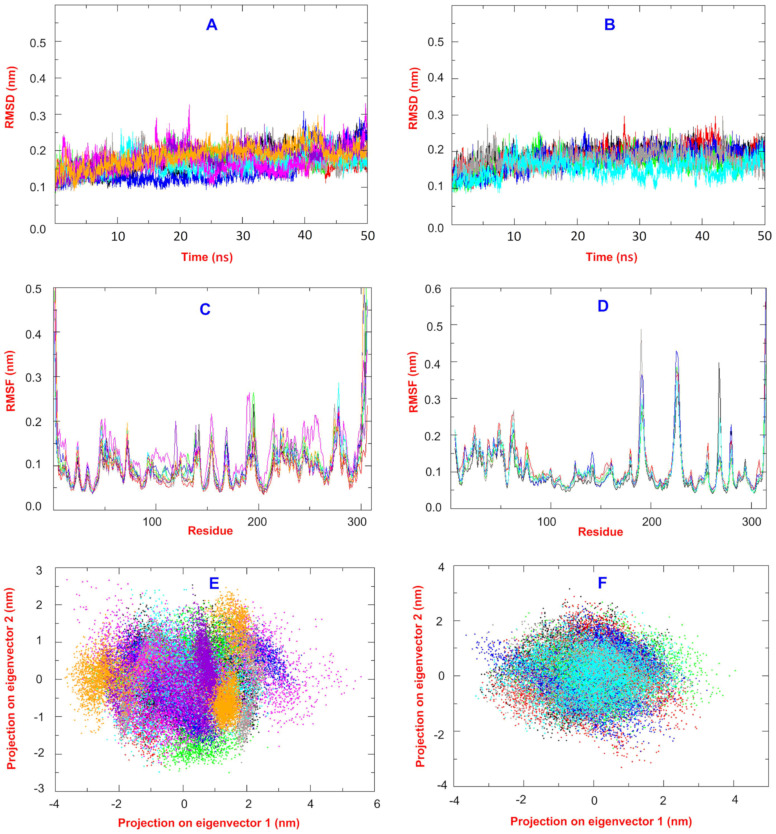
(**A**,**B**) RMSD plots for Cα variations during the MD simulation time 50 ns for the 3CLpro and PLpro complexes, respectively. (**C**,**D**) RMSF plots for Cα variations during the MD simulation time of 50 ns for the 3CLpro and PLpro complexes, respectively. (**E**,**F**) 2D projection of the first and second eigenvectors for the 3CLpro and PLpro complexes, respectively. For the 3CLpro complexes: black: baicalin, red: CHA-12, green: CHA-384, blue: CHA-37, brown: CHA-297, cyan: CHA-378, magenta: hirsutenone, violet: compound A, and orange: compound B. For the PLpro complexes: black: co-crystal ligand, red: GRL0617, green: CHA-12, blue: CHA-37, brown: CHA-378, and cyan: hirsutenone.

The best hits found by docking virtual screening were chosen for further molecular dynamics and MM/PB(GB)SA analysis. Baicalein as a co-crystal ligand and a standard inhibitor of the 3CLpro and GRL0617 as a well-known and standard inhibitor of the PLpro were considered in our study. Hirsutenone was also used in our computational and structural analysis as an experimentally reported 3CLpro and PLpro inhibitor (Figure 3). Correspondingly, CHA-12, CHA-384, CHA-378, CHA-297, CHA-37, and compounds A and B were studied in our molecular dynamics and binding free energy evaluations in the 3CLpro active site. Similarly, ligands CHA-12, CHA-37, and CHA-378 were used in our molecular dynamics and binding free energy calculations in the PLpro active site (Figure 3). An overlay of all the RMSD plots of the simulations with the 3CLpro and PLpro with respect to the C-alpha were calculated and are shown in Figure 4A,B, respectively. RMSD plot is a useful index to evaluate the stability of the protein and the complex during the simulation time. Totally, the convergence of the RMSD curve around a smaller value indicates the favorability and high stability of the complex.

As seen in Figure 4A,B, all the 3CLpro and PLpro-ligand complexes have minor variations and oscillations during the simulation time of 50 ns which are ending in the range of 0.15–0.25 nm at the end of the simulation and, accordingly, all are in very stable equilibrated conditions in the whole simulation time.

An overlay of all the RMSF plots of the simulations with 3CLpro and PLpro with respect to the C-alpha were calculated and are shown in Figure 4C,D, respectively. The RMSF curve of the simulation shows the flexibility and dynamics of the protein main chain corresponding to each amino acid residue. Totally, the minimal fluctuations over the entire amino acid sequence indicate a stable structure, although flexible regions of the protein such as loops and the two ends of the protein normally have greater values of fluctuations compared to the internal parts and structured alpha-helix or beta-sheets. Overlay of all RMSF plots of the 3CLpro and PLpro shows that the average value for all the complexes is around 0.10 nm, and the stability of complexes is in good accordance with the RMSD values. As seen in Figure 4C, the RMSF curves of all ligands in the 3Clpro have been superimposed well on each other with minimal differences and clearly confirm high integrity and normal conditions for all dynamic simulations. The same fact also exists for all ligands equilibrated in the PLpro as shown by their well-superimposed RMSF curves (Figure 4D).

All the RMSD and RMSF plots together confirmed the minor geometrical and structural changes during the simulation time and the conservation of the initial crystal structure of the protein before and after the dynamic process. The trajectories of MD simulations were used for the covariance analysis in order to evaluate the dynamic motions of the protein. A 918*918 covariance matrix based on the carbon-alpha fluctuations was diagonalized to generate eigenvalues against the eigenvectors, and the first 50 eigenvectors showed ≥ 99% of the variance of motions for each dynamic simulation. Accordingly, a 2D projection of the two first principal components PC1 and PC2 that represent the dominant conformational fluctuations for all the ligands studied in the 3CLpro and PLpro active sites are visualized in Figure 4E,F, respectively. The results obtained by covariance analysis are in good agreement with the RMSD and RMSF plots. Trace values of the covariance matrix before and after the diagonalizing are between 3.06 and 4.13 for all ligands studied in the 3Clpro.

The trace values of the covariance matrix before and after the diagonalizing for ligands studied in the PLpro are in the range of 3.69–5.48 (Figure 4F).

**Figure 5:**
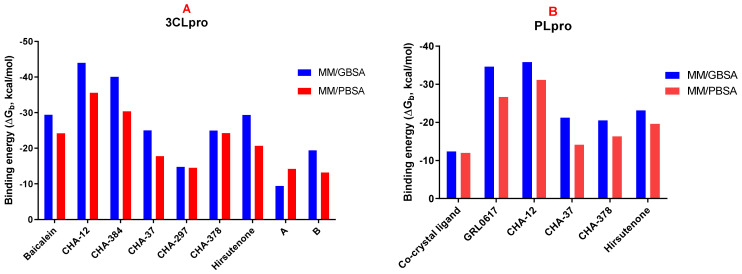
Binding free energy (ΔG_b_) of compounds calculated by the MM/GBSA (blue) and MM/PBSA (red) methods in the active sites of the 3CLpro (**A**) and PLpro (**B**).

In order to calculate the binding free energy of the selected hit compounds, the last trajectories of the MD simulations were used for the Molecular Mechanics/Poisson Boltzmann (or Generalized Born) Surface Area (MM/PB(GB)SA) analysis. Final results of the MM/PB(GB)SA analysis as binding free energy (ΔG_b_ in kcal/mol) are visualized in Figure 5A,B for the 3CLpro and PLpro, respectively. Values of the energy components, total free energy (ΔG_total_), solvation energy (ΔG_solv_), gas-phase free energy (ΔG_gas_), and the interaction entropy (IE) for each compound studied in MD and MM/PB(GB)SA analysis along with their final binding free energy values (ΔG_b_) are summarized in Table 3 and Table 4 for the 3CLpro and PLpro, respectively.

As shown in Figure 5A, CHA-12 has the best binding affinity towards the 3CLpro among the compounds studied in our library with the binding free energy of −44.03 and −35.57 kcal/mol, respectively, calculated by the MM/GBSA and MM/PBSA methods. Interestingly, CHA-12 has even more binding affinity than the 3CLpro co-crystal ligand baicalein (ΔG_b_ = −29.43 and −24.23 kcal/mol calculated by the MM/GBSA and MM/PBSA, respectively), and it is also better than the well-known 3CLpro inhibitor hirsutenone (ΔG_b_ = −29.37 and −20.66 kcal/mol calculated by the MM/GBSA and MM/PBSA methods, respectively) in the active site of the 3CLpro.

CHA-384 which is a hybrid of a chalcone and ureide moiety is in the second order of affinity towards the 3CLpro (ΔG_b_ = −40.05 and −30.39 kcal/mol, respectively, calculated by the MM/GBSA and MM/PBSA methods) and showed better binding affinity than baicalein and hirsutenone (in both MM/GBSA and MM/PBSA methods). Based on the results, CHA-384 was found and considered to be a selective 3CLpro inhibitor among the 757 chalcone-based compounds evaluated in this study.

The sulfonamide-containing compound CHA-378 with ΔG_b_ = −24.98 and −24.28 kcal/mol, respectively, calculated by the MM/GBSA and MM/PBSA methods was shown to have superior affinity towards 3CLpro than its congener CHA-297. CHA-378 showed more potency than hirsutenone with ΔG_b_ = −20.66 and was found to be equivalent to baicalein with ΔG_b_ = −24.23 in MM/PBSA calculations.

Results of the MM/GBSA and MM/PBSA calculations indicated that CHA-37 has a weaker affinity towards 3CLpro than baicalein and hirsutenone.

Fragment compounds A and B bearing sulfonamide and ureide moieties, respectively, did not show acceptable affinity due to their smaller size but were included in our study to indicate their site-specific occupancy in the respective active site (Table 3).

Interestingly, CHA-12 was also shown to have the best affinity towards PLpro calculated by both MM/GBSA and MM/PBSA methods with ΔG_b_ = −35.81 and −31.13 kcal/mol, respectively. It was found that this compound is more potent than the well-known PLpro selective inhibitor GRL0617 (ΔG_b_ = −34.65 and −26.64 kcal/mol by MM/GBSA and MM/PBSA, respectively) and hirsutenone (ΔG_b_ = −23.11 and −19.61 kcal/mol by MM/GBSA and MM/PBSA, respectively). Ligands CHA-378 with ΔG_b_ = −20.52 and −16.31 and CHA-37 with ΔG_b_ = −21.23 and −14.11 kcal/mol calculated by MM/GBSA and MM/PBSA, respectively, were not found to have better affinity than GRL0617 and hirsutenone towards PLpro but had more potency than the co-crystalized ligand (Table 4).

**Figure 6:**
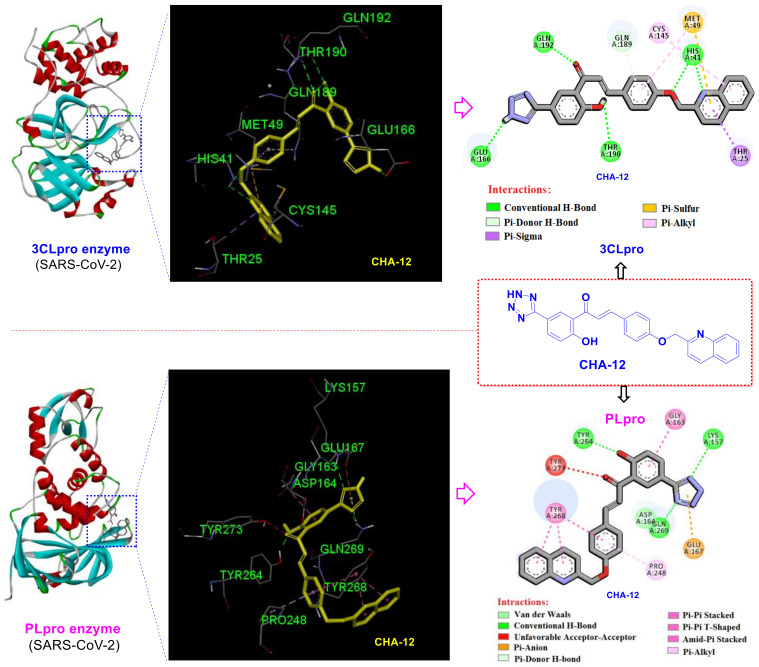
2D and 3D interactions of CHA-12 in the active site of the SARS-CoV-2 3CLpro and PLpro enzymes.

Results of the current study revealed that the hit compound CHA-12 has a magic affinity towards all tested targets. The most important interactions of CHA-12 in the active site of 3CLpro are shown in Figure 6. Totally, hydrogen bonds were found to be the main part of the binding affinity. Essentially, several hydrogen bondings were observed between the bound conformation of CHA-12 and the 3CLpro active site residues. In fact, two hydrogen bonds are formed by the interaction of His41 with the quinoline nitrogen and oxygen atom of the oxymethylene chain; one hydrogen bond exists between Glu166 and the NH-tetrazole, another hydrogen bonding between Thr190 and phenolic hydroxyl group, and, finally, one hydrogen bonding also exists between Gln192 and the carbonyl oxygen atom. In addition, Pi-sulfur interactions of Met49 to the quinolone and phenyl rings, a Pi-sigma interaction of Thr25 with the pyridine ring of quinoline moiety, and a Pi-donor H-bond interaction of Gln189 with the phenyl ring of the chalcone backbone contribute to the stronger binding of CHA-12 in the 3CLpro active site.

Evaluation of the CHA-12 interactions at the PLpro active site shows that the tetrazole moiety forms two key hydrogen bonds with Lys157 and Gln269 residues, as well as a Pi-anion interaction with the Glu167. A hydrogen bonding also exists through phenolic hydroxyl group with Tyr264 and Tyr268 has three interactions of type Pi-Pi stacked, Pi-Pi T-shaped, and amide-Pi stacked with the phenyl and quinoline moieties of CHA-12. There is also a Pi-alkyl interaction between Pro248 and the phenyl ring of CHA-12 (Figure 6). Therefore, reported relaxant effects of CHA-12 on pulmonary smooth muscles established by in vitro and in vivo assays [47], along with its prominent predicted inhibitory effects on the both SARS-CoV-2 targets, supports the discovery of a valuable compound for the treatment of COVID-19.

The compound CHA-378 bearing a sulfonamide moiety showed acceptable results in the active site of the 3CLpro and PLpro targets. The CHA-378 generates strong interactions in the active sites of most HBATs including p38 MAPK (rank 10, docking score: −9.0 kcal/mol), cathepsin L (rank 10, docking score: −8.0 kcal/mol), CDK2/CyclinA (rank 5, docking score: −10.1 kcal/mol), CDK9/cyclinT1 (rank 4, docking score: −10.4 kcal/mol), RBD2 (rank 1, docking score: −9.4 kcal/mol), RBD4 (rank 4, docking score: −9.8 kcal/mol), and both SARS-CoV-2 targets 3CLpro (rank 4, docking score: −8.7 kcal/mol) and PLpro (rank 10, docking score: −9.1 kcal/mol) (Table 2 and Supplementary Excel File related to the docking scores).

**Figure 7:**
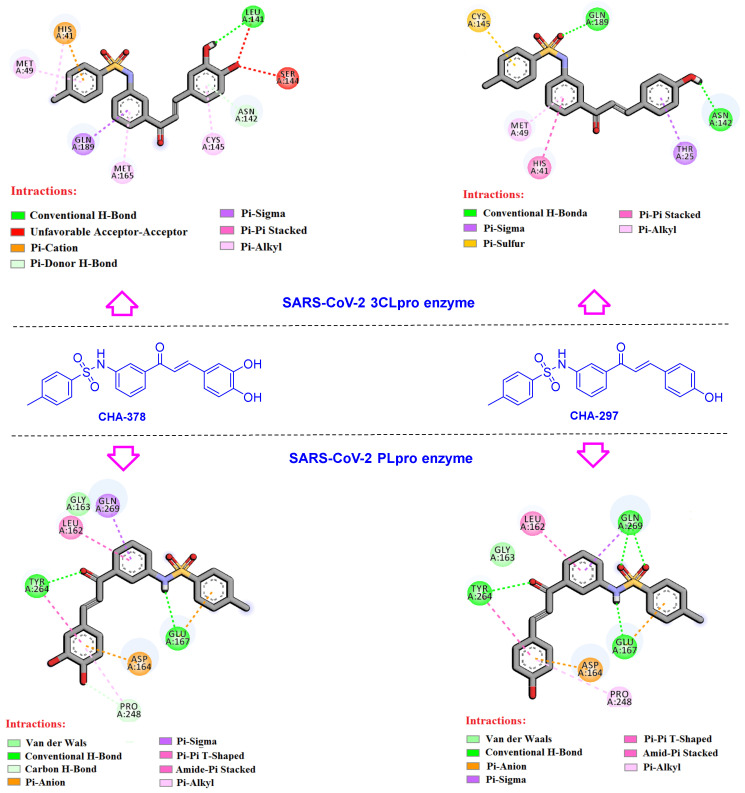
Interactions of CHA-297 and CHA-378 in the active site of the SARS-CoV-2 3CLpro and PLpro enzymes.

The types and interacting residues for the CHA-378 and its analogue CHA-297 in the active site of PLpro and 3CLpro are displayed in Figure 7 and Tables S4 and S5. The key interactions created by CHA-378 at the 3CLpro active site are as follows: a conventional hydrogen bond (Leu141 with 3-OH of catechol ring), a Pi-donor H-bond (Asn142 with catechol ring), four Pi-alkyl interactions (Met49, Met165, Cys145, and His41), Pi-sigma interaction (Gln189), and two Pi-cation and Pi-Pi stacked interactions of His41 with a phenyl ring of sulfonamide moiety. There is also an unfavorable acceptor–acceptor interaction of Ser144 and Leu141 with the 4-hydroxy group of the catechol moiety.

Interestingly, it was found that the sulfonamide moiety occupied the S3 subsite and hydrogen bonded to Gln189, which has been proved by a recent study to be a specific site for the binding of the sulfonamide containing 3CLpro inhibitors [10].

Compounds CHA-378 and CHA-297 were also investigated in the active site of the PLpro since the primary results of the docking screening indicated them as PLpro inhibitors as well (Table 2). In summary, NH of the sulfonamide moieties in both CHA-378 and CHA-297 creates a hydrogen bond with Glu167 in the active site of the PLpro. On the other hand, oxygen atoms of the sulfonamide moiety of CHA-297 formed hydrogen bond interactions with Gln269. Similarly, Gln269 and Leu162 residues formed the same interactions, Pi-sigma (Gln269) and amide-Pi stacked (Leu162), with the A-ring of the chalcone backbone in both CHA-297 and CHA-378. Tyr264 is another key residue that creates the same interactions in the PLpro active site for both CHA-297 and CHA-378, including a conventional hydrogen bond with the carbonyl group of the chalcone moiety, and a Pi-Pi T-shaped interaction with the catechol and phenolic rings of CHA-378 and CHA-297, respectively.

Among the evaluated 3CLpro-selective ureide-chalcones, CHA-384 generates the highest affinities in the active site of the virus and host-based targets compared to the other congeners. The rankings predicted for CHA-384 against the different virus and host-based targets are as follows (see Table 2 and Supplementary Excel File related to the docking scores): CDK2/CyclinA (rank 7, docking score: −9.2 kcal/mol), ERK2 (rank 2, docking score: −9.9 kcal/mol), DHODH (rank 2, docking score: −12.7 kcal/mol), BRD4 (rank 7, docking score: −9.7 kcal/mol), sigma-1 receptor (rank 3, docking score: −11.6 kcal/mol), and 3CLpro (rank 2, docking score: −8.9 kcal/mol). In addition to the conventional hydrogen bonds mentioned above, the most important interactions of CHA-384 in the active site of the 3CLpro are as follows: Pi-Alkyl interactions of Met49 and Met165 residues with A-phenyl ring of the chalcone backbone and Pi-Alkyl interaction of Pro168 with phenyl ring of the ureide moiety. Moreover, the chlorine atom of the ureide moiety generates two alkyl interactions with Pro168 and Ala191 residues. The Pi-sigma interaction of the B-phenyl ring with Thr25, and a Pi-Pi stacked interaction of the A-phenyl ring with His41 are other interactions formed by CHA-384 in the active site of the 3CLpro. These interactions are discussed comparatively in more detail for CHA-384 and its congeners in Section 3.3.

Among our chalcone-based compound library, CHA-7, CHA-11, CHA-37, CHA-44, and CHA-177 showed superior docking scores against the SARS-CoV-2 PLpro and host-based targets, and CHA-37 was found to have the highest affinity towards the PLpro among them (see Table 2 and Supplementary Excel File related to the docking scores). The ranking and docking energy score of CHA-37 at the active sites of the host and viral-based targets are as follows: p38 MAPK (rank 2, −9.4 kcal/mol), Cathepsin L (rank 4, −8.3 kcal/mol), CDK1 (rank 3, −9.3 kcal/mol), CDK2/CyclinA (rank 9, −9.6 kcal/mol), CDK9/cyclinT1 (rank 7, −9.9 kcal/mol), ERK2 (rank 4, −9.5 kcal/mol), DHODH (rank 9, −12.0 kcal/mol), CK2 alpha’ (rank 1, −11.4 kcal/mol), BRD2 (rank 5, −8.8 kcal/mol), BRD4 (rank 1, −10.4 kcal/mol), and sigma-1 receptor (rank 2, −11.7 kcal/mol), 3CLpro (rank 12, −8.3 kcal/mol), and PLpro (rank 3, −9.5 kcal/mol). Although the calculated binding affinity of CHA-37 in the active site of the PLpro was shown to be lower than GRL0617 by the MM/PBSA and MM/GBSA methods, it still shows more affinity than the co-crystal ligand of the 7JN2 PDB code (Table 4).

**Figure 8:**
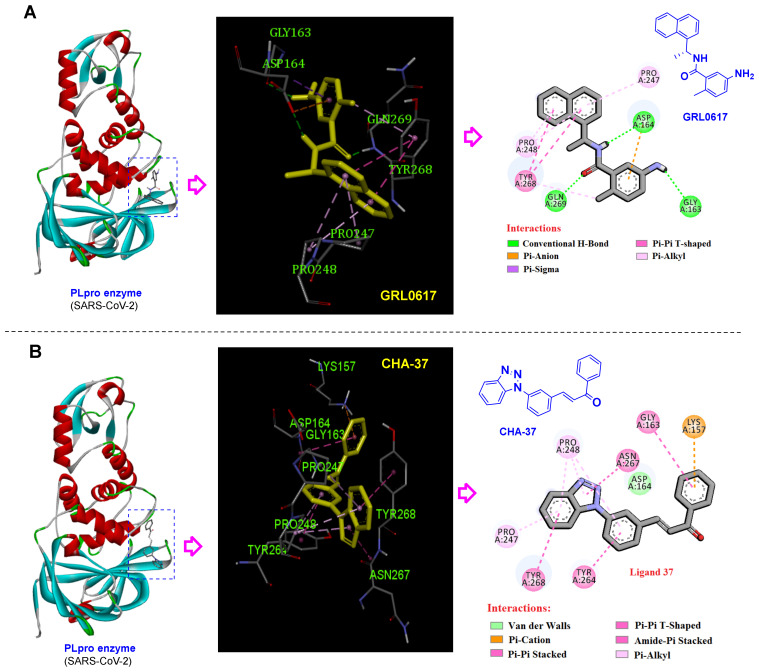
2D and 3D interactions of (**A**) GRL0617 (as a standard SARS-CoV-2 PLpro inhibitor) and (**B**) **CHA-37** in the active site of the SARS-CoV-2 PLpro enzyme.

As displayed in Figure 8B and Table S5, CHA-37 created important interactions in the active site of the PLpro. The benzotriazole moiety of this compound forms key interactions, including Pi-alkyl with Pro247 and Pro248 residues, one amide-Pi stacked interaction resulting from the interaction of triazole fraction with Asn267, and one Pi-Pi T-shaped interaction with Tyr268. Similarly, the naphthyl ring of the GRL0617 creates three Pi-alkyl interactions with Pro247 and Pro248 residues, and also two Pi-Pi T-shaped interactions with Tyr268. In addition, the formation of three conventional hydrogen bonds by the carbonyl group with Tyr268, amide-NH with Asp164, and the amino group of GRL0617 with Gly163 help to strengthen the binding of this compound in the active site of the SARS-CoV-2 PLpro. 2D and 3D interactions of CHA-37 and GRL0617 in the active site of the SARS-CoV-2 PLpro enzyme are shown in Figure 8.

### 2.3. Druglikeness, ADME, and Toxicity

Natural-based compounds quercetin and resveratrol are among the most important safe supplements suggested to combat COVID-19 as adjuvants [48,49,50]. Due to their valuable medicinal effects, these compounds have long been available in the market as oral supplements. Herein, the drug-likeness, ADME properties, oral bioavailability, and hepatotoxicity of the most important identified compounds CHA-12, CHA-37, CHA-378, and CHA-384 are compared to the quercetin and resveratrol as standard medicines. Based on the results of these evaluations, it is predicted that the most active compounds identified in this study have comparable or even better drug-likeness profiles compared to the standard compounds quercetin and resveratrol (Table S2).

#### 2.3.1. Oral Bioavailability

Oral bioavailability is often an important consideration for the discovery and development of bioactive molecules as therapeutic agents. Thus, the understanding of the molecular properties that limit oral bioavailability is an important goal for drug research. The most important parameters that are considered for the evaluation of oral bioavailability include lipophilicity, molecular size, molecular polarity, insolubility, insaturation of molecular structure, and molecular flexibility. As shown in Figure S1, of the six parameters considered, CHA-12, CHA-37, CHA-384, and CHA-378 have five parameters in the desired range. Accordingly, these compounds are expected to have appropriate oral bioavailability.

#### 2.3.2. Cytochrome P450 Interaction

Hepatotoxicity is one of the most important issues in the process of discovering new drugs. The effects of the evaluated compounds on increasing or decreasing the function of cytochrome P450 enzymes have always been considered [51]. CYP3A4, CYP2D6, CYP2C9, and CYP2C19 isoforms are the most well-known subtypes of liver cytochrome P450 enzymes that play a vital role in drug metabolism and their effective or toxic concentrations in the body [52]. Because of the importance of this issue, we have tried to predict the effects of CHA-12, CHA-37, CHA-378, CHA-384, resveratrol, and quercetin on the liver enzymes, using computational methods. According to the results in Table S3, unlike standard drugs quercetin and resveratrol which inhibit CYP2C19 and CYP3A4, CHA-12, CHA-37, and CHA-384 are not expected to have inhibitory effects on these enzymes. In addition, the effects of these ligands on the other cytochrome P450 enzymes (CYP2C9, CYP2D6, and CYP3A4) are similar to the effects of the standard drugs. Therefore, these ligands are predicted to have fewer hepatotoxic effects than the standard drugs.

## 3. Discussion

Comparison of the calculated ΔG_b_ of the standard inhibitors baicalein, hirsutenone, and GRL0617 with their experimental affinities towards the 3CLpro and PLpro clearly indicates that the results of the MM/PB(GB)SA computations are reliable. Baicalein as a standard inhibitor of the SARS-CoV-2 3CLpro has shown high affinity with the calculated binding energy of −29.43 kcal/mol in the MM/GBSA analysis, which is consistent with its experimentally reported IC_50_ value of 0.39 µM. Correspondingly, GRL0617 as a standard inhibitor of the SARS-CoV-2 PLpro with calculated ΔG_b_ = −34.65 and −26.64 kcal/mol by MM/GBSA and MM/PBSA methods, respectively, indicates its high affinity towards the enzyme and is fully consistent with its experimentally reported IC50 value of 1.6 µM.

**Figure 9:**
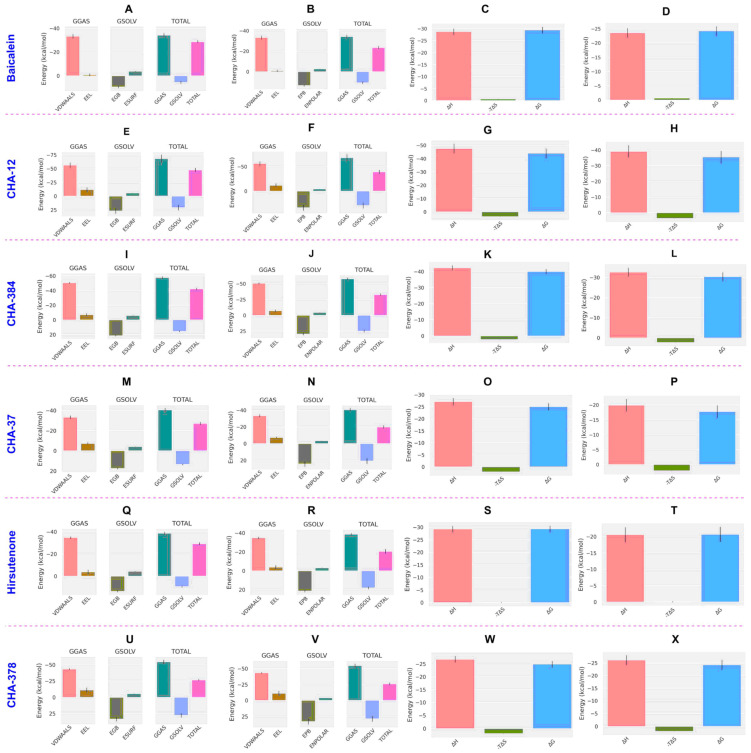
Energy components of the compounds calculated by the MM/GBSA and MM/PBSA in the 3CLpro. Enthalpic components of baicalein in MM/GBSA (**A**) and MM/PBSA (**B**) analysis and its binding free energy (ΔG_b_) decomposed to the enthalpy (ΔH) and entropy (-TΔS) terms in the MM/GBSA (**C**) and MM/PBSA (**D**) calculations. Correspondingly, enthalpic components and binding free energy (ΔG_b_) terms for CHA-12 in the MM/GBSA and MM/PBSA calculations are respectively illustrated in (**E**–**H**). Enthalpic components and binding free energy (ΔG_b_) terms for CHA-384 in the MM/GBSA and MM/PBSA calculations are respectively illustrated in (**I**–**L**). Enthalpic components and binding free energy (ΔG_b_) terms for CHA-37 in the MM/GBSA and MM/PBSA calculations are respectively illustrated in (**M**–**P**). Enthalpic components and binding free energy (ΔG_b_) terms for hirsutenone in the MM/GBSA and MM/PBSA calculations are respectively illustrated in (**Q**–**T**). Enthalpic components and binding free energy (ΔG_b_) terms for CHA-378 in the MM/GBSA and MM/PBSA calculations are respectively illustrated in (**U**–**X**). VDWAALS: van der Waals energy, EEL: electrostatic energy, EGB: polar solvation energy calculated by the MM/GBSA, ESURF: non-polar solvation energy calculated by the MM/GBSA, EPB: polar solvation energy calculated by the MM/PBSA, ENPOLAR: non-polar solvation energy calculated by the MM/PBSA.

Energy components of binding clearly indicate that the high binding affinity of CHA-12 is reasoned by its favorable interaction entropy (IE) of 0.04 and its high enthalpic contribution represented by its ΔG_gas_ of −68.26 kcal/mol (Table 3). The diagram of the energy components shown in Figure 9E,F clearly indicates that the van der Waals and electrostatic energies are more favored by CHA-12 than baicalein and CHA-384. Ortho-substitution of the phenyltetrazole ring by hydroxyl and ketone groups facilitates the formation of an intra-hydrogen bond between the oxygen atom of the ketone and the proton of the hydroxyl group. This phenomenon restricts the free rotation of the chalcone linker and favors the entropy cost upon binding. Diagrams of the energy components constituting the ΔG_T_ and ΔG_b_ of baicalein, CHA12, CHA-384, CHA-37, and hirsutenone in the active site of the 3CLpro are represented in Figure 9.

**Figure 10:**
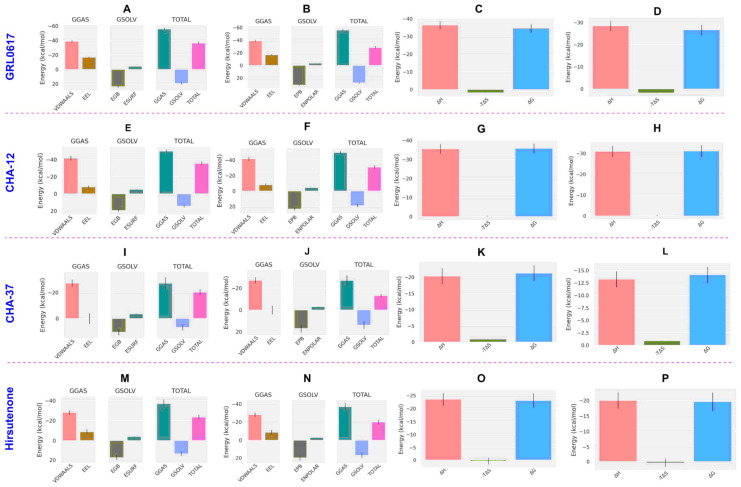
Energy components of the compounds calculated by the MM/GBSA and MM/PBSA in the PLpro. Energy components of GRL0617 in the MM/GBSA (**A**) and MM/PBSA (**B**) analysis and its binding free energy (ΔG_b_) decomposed to the enthalpy (ΔH) and entropy (-TΔS) terms in the MM/GBSA (**C**) and MM/PBSA (**D**) calculations. Correspondingly, energy components and binding free energy (ΔG_b_) terms for CHA-12 in the MM/GBSA and the MM/PBSA calculations are respectively illustrated in (**E**–**H**). Energy components and binding free energy (ΔG_b_) terms for CHA-37 in the MM/GBSA and MM/PBSA calculations are respectively illustrated in (**I**–**L**). Energy components and binding free energy (ΔG_b_) terms for hirsutenone in the MM/GBSA and MM/PBSA calculations are respectively illustrated in (**M**–**P**). VDWAALS: van der Waals energy, EEL: electrostatic energy, EGB: polar solvation energy calculated by the MM/GBSA, ESURF: non-polar solvation energy calculated by the MM/GBSA, EPB: polar solvation energy calculated by the MM/PBSA, ENPOLAR: non-polar solvation energy calculated by the MM/PBSA.

In the case of the PLpro, CHA-12 was again found to be the most potent compound calculated by the MM/PB(GB)SA method. CHA-12 with binding free energy (ΔG_b_) of −35.81 and −31.13 kcal/mol, respectively, calculated by the MM/GBSA and MM/PBSA methods was much better than the standard inhibitor GRL0617 (ΔG_b_ = −34.65 and −26.64 kcal/mol, respectively, calculated by the MM/GBSA and MM/PBSA methods) and hirsutenone (ΔG_b_ = −23.11 and −19.61 kcal/mol, respectively, by the MM/GBSA and MM/PBSA) in the active site of the PLpro. Enthalpic contributions including van der Waals and electrostatic energies are the main factors favoring the total binding energy of CHA-12 as represented in Table 4 and Figure 10. In addition, the interaction entropy cost of CHA-12 is a small quantity of +0.16 kcal/mol. Therefore, CHA-12 is considered a multi-functional compound affecting various virus and host-based biological pathways as discussed earlier in the virtual screening results of the docking scores of host-based targets. Ligand CHA-37 was shown to be a higher affinity PLpro inhibitor than the co-crystalized ligand, which is indicated by the MM/PBSA and MM/GBSA results (CHA-37, ΔG_b_ = −21.23; co-crystalized ligand, ΔG_b_ = −12.36 kcal/mol). CHA-37 has fewer van der Waals and electrostatic energies than CHA-12 as shown in Figure 10. CHA-37 also has a very small entropy cost of 0.04 kcal/mol. The sulfonamide-containing compound CHA-378 was found to be a weak binder for the PLpro active site compared to CHA-12 and the standard inhibitors GRL0617 and hirsutenone. This weak binding affinity of CHA-378 was also observed for the 3CLpro as discussed earlier.

### 3.1. CHA-12 as a Multi-Target Inhibitor

Leukotrienes are broncho-constrictors whose pharmacological effects mimic the pathological changes in asthma. The biological activity of leukotrienes is mediated by the activation of cysteine leukotrienes (CysLT1 and CysLT2), leading to bronchoconstriction, mucus secretion, and asthma-like conditions. Accordingly, CysLT1 (LTD4) receptor antagonists are considered important therapeutic agents in the treatment of lung and respiratory diseases such as asthma. Interestingly, CHA-12 was first introduced as a CysLT1 antagonist in 1997 in a study by Mariel E. Zwaagstra et al. [47]. Among more than sixty compounds synthesized in that study, CHA-12 showed the best CysLT1 antagonistic activity in both in vitro and in vivo assays. In fact, patients infected with COVID-19 suffer from acute inflammation in the lung and respiratory system mostly due to the cytokine storm, and having this important pharmacological activity of CHA-12 greatly increases the chance of CHA-12 as a lead compound for treating COVID-19 complications in the respiratory system.

**Figure 11:**
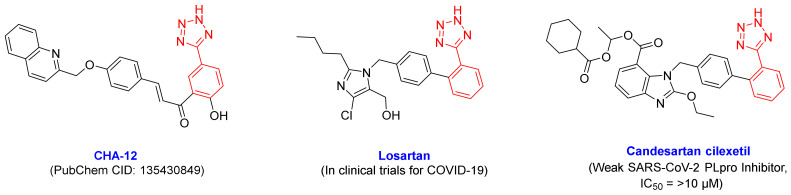
Structural similarity of CHA-12, losartan, and candesartan cilexetil due to the existence of a phenyltetrazole moiety.

Impressive results of the virtual screening of the 757 chalcone-based compound library over 14 host- and virus-based molecular targets, and also the MM/PB(GB)SA analysis, indicated that CHA-12 is a unique compound and has the highest affinity towards the 3CLpro and PLpro active sites. As shown in Figure 6, CHA-12 fits well into the 3CLpro active site. Paying attention to the structure of this compound reminds us of interesting similarities with some well-known drugs. The presence of the phenyltetrazole moiety in the structure of this compound structurally makes a partial similarity to losartan, an angiotensin II receptor blocker (ARB) reported to have a remarkable effect on the treatment of COVID-19 (Figure 11) [53,54,55,56,57]. In a recent HTS study performed by Zhengnan Shen et al., candesartan as an ARB was identified as one of a few compounds that showed inhibitory effects against SARS-CoV-2 PLpro out of about 15,000 screened compounds [58]. The phenyltetrazole moiety of CHA-12 makes crucial interactions in the active site of the viral targets (especially PLpro), which are discussed later in the following.

**Figure 12:**
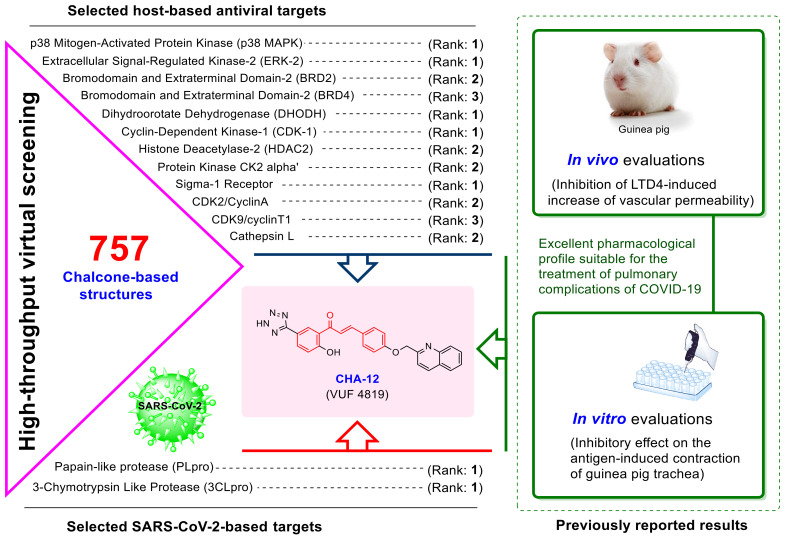
A summary of the remarkable potentials of CHA-12 to combat SARS-CoV-2 infection as well as to reduce the pulmonary complications of COVID-19. The excellent relaxant effects of CHA-12 on pulmonary smooth muscle through antagonistic activity on CysLT1 (proven in vivo and in vitro), along with the prediction of remarkable effects on all host-based and SARS-CoV-2 3CLpro and PLpro targets, promise the discovery of a unique compound with high potential for the treatment of COVID-19.

Overall, CHA-12 was discovered to be the most potent and multi-targeted inhibitor in our chemical library against all host- and virus-based molecular targets. Since this compound has also shown the best in vitro and in vivo activity among the chalcone compounds tested in past laboratory studies, this finding prompts us to introduce CHA-12 as a concomitant compound to relieve respiratory symptoms of COVID-19 infection. Figure 12 schematically summarizes the significant potential of this compound to fight the infection of SARS-CoV-2 and also to reduce the pulmonary complications of COVID-19.

### 3.2. Chalcones Bearing Sulfonamide Moiety

**Figure 13:**
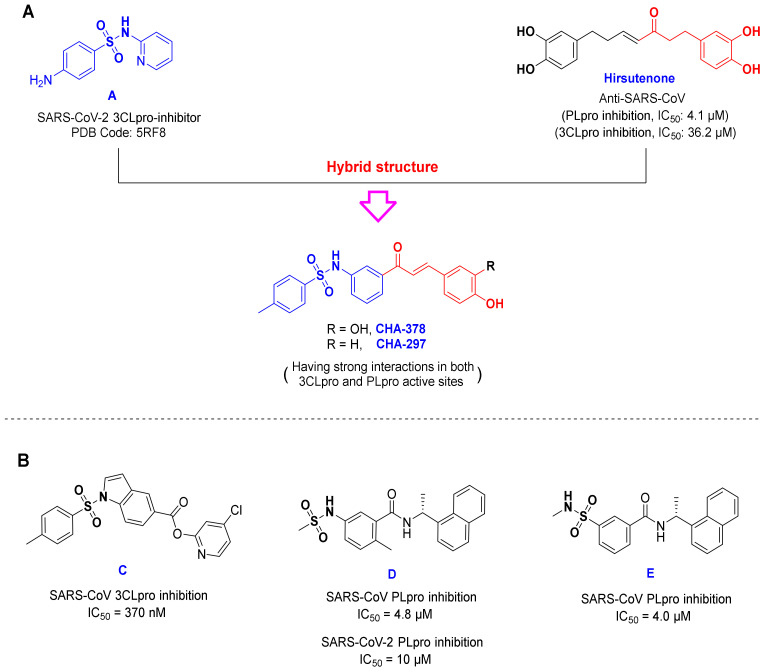
(**A**) The chemical structure of CHA-297, CHA-378 and its structural similarity with compound A and hirsutenone. (**B**) Structures of compounds **C** **D** and **E**.

The compounds CHA-378 and CHA-297 bearing a sulfonamide moiety are also interesting compounds and showed relatively acceptable results in the active site of the 3CLpro and PLpro targets. Structurally, CHA-378 consists of two structural moieties: a chalcone scaffold and a moiety containing sulfonamide functional group. This compound seems to be an interesting hybrid of hirsutenone (a well-known SARS-CoV 3CLpro and PLpro inhibitor [59]) and compound A (Figure 13). The latter was first synthesized in 2005 by Seo et al. as an α-glucosidase inhibitor. In their study, among all synthesized compounds, this ligand showed the best α-glucosidase inhibitory activity with IC_50_ = 0.4 µM [60]. Interestingly, in a recent study, Williams and Borger highlighted α-glucosidase inhibitors as host cell N-glycosylation pathway blockers with high potential for the treatment of COVID-19 [61].

In a recently published study, a large-scale screening of electrophile and non-covalent fragments was performed by Douangamath et al. against the SARS-CoV-2 3CLpro through combined mass spectrometry (MS) and X-ray method [10]. Evaluations of this study showed that the active site of the SARS-CoV-2 3CLpro has four subsites namely S1, S1’, S2, and S3. The S3 subsite has been reported to be explored and occupied by the sulfonamide group of the inhibitors and interacts with hydrogen bonding to Gln189. This space consists of His164, Met165, and Asp187, which is exposed to the solvent. Therefore, the fragments located in this space can be oriented to the solvent. The crystal structure of 3CLpro protein bound to compound A has recently been released in the Protein Data Bank with the PDB Code of 5RF8. This compound is able to create specific interactions in the S3 subsite. In a combined X-ray crystallography and fragment screening method, Charlie Nichols et al. reported that this fragment could interact well in the active site of the P38 MAPK target (PDB code: 5R9P) [62]. The presence of a structural moiety similar to this fragment (compound A) in the structure of CHA-378 (Figure 13) can be one of the important reasons for creating remarkable interactions in the active site of the p38 MAPK target (rank 10, −9.0 kcal/mol) in our screening results. Moreover, p38 MAPK was recently highlighted as one of the most promising targets for the treatment of COVID-19 [23].

The sulfonamide moiety has frequently been found in the structure of both the reported 3CLpro and PLpro inhibitors. In 2008, Ghosh and co-workers developed small molecules with potent inhibitory activity against SARS-CoV 3CLpro, some of which contain the sulfonamide functional group (Figure 13, compound C) [63]. In a recently published study, Welker and co-workers also introduced some chalcone-amide-based small molecules with inhibitory activities against SARS-CoV/SARS-CoV-2 PLpro, which contain a sulfonamide moiety (Figure 13, compounds D and E) [64]. The mentioned experimental results together with our computational findings indicate that compounds CHA-297 and CHA-378 could be specific compounds with the remarkable potential to inhibit SARS-CoV-2.

### 3.3. Active Ligands Selective towards the 3CLpro

**Figure 14:**
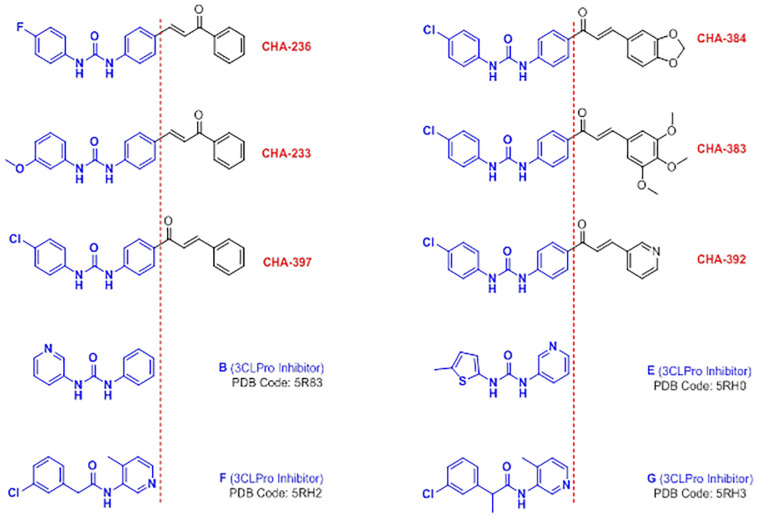
Structural similarity of the identified selective-3CLpro ligands CHA-233, CHA-236, CHA-383, CHA-384, CHA-392, and CHA-397, the recently reported biaryl urea compounds B and E, and their isosteric amide-analogue compounds F and G as special fragments introduced for the design and development of 3CLpro-inhibitors.

The biphenyl urea scaffold is a recently identified fragment introduced by Douangamath and co-workers that can generate specific interactions in the active site of the SARS-CoV-2 3CLpro enzyme [10]. As the researchers well characterized it, the S1 subsite of the 3CLpro-active site revealed the two key residues His163, which is interacting with the pyridine nitrogen or with nitrogen-containing heterocycles, and Glu166, which is a key amino acid in the formation of hydrogen bonds to the amide or ureide substructures of the 3CLpro-inhibitors. In Figure 14, the chemical structures of some of the biaryl urea small molecules (compounds B and E) and their isosteric amide-analogues (compounds F and G) with their corresponding PDB codes, which were recently released as selective 3CLpro inhibitors, are displayed. In addition, the structural similarity of the reported biaryl urea compounds with some of our ureide-chalcone hybrid structures existed in our library which were found to be selective against 3CLpro (see Table 2) is displayed in Figure 14.

**Figure 15:**
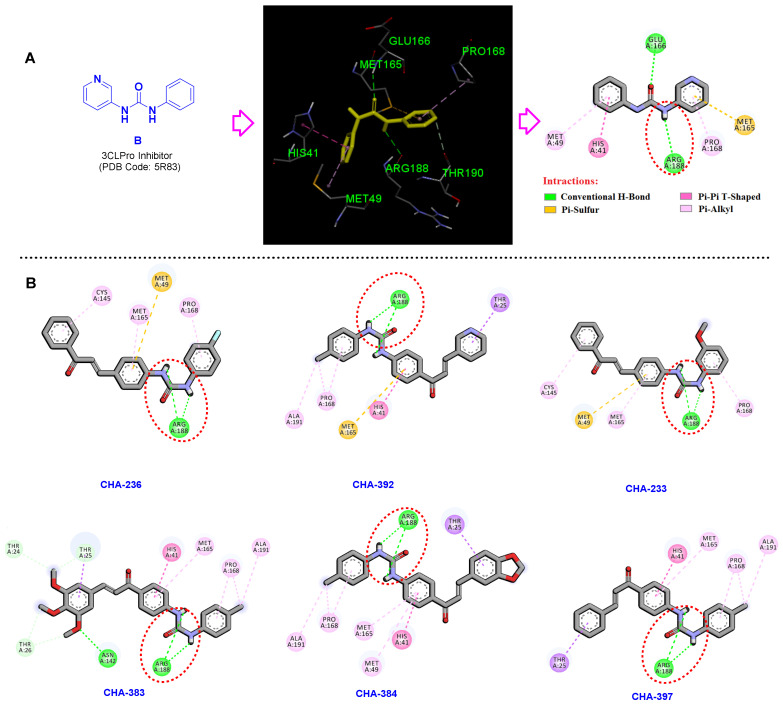
(**A**) Compound **B** in the active site of the 3CLpro. (**B**) Establishment of the two hydrogen bonds formed in the active site of the SARS-CoV-2 3CLpro by the ureide-chalcone hybrid structures CHA-233, CHA-236, CHA-383, CHA-384, CHA-392, and CHA-397 and the carbonyl group of Arg188.

The key interactions of the co-crystal compound B in the active site of the 3CLpro (PDB code 5R83) are shown in Figure 15A. Correspondingly, NH and oxygen atoms of the ureide moiety in compound B form two important conventional hydrogen bonds with the residues Arg188 and Glu166. Investigating the binding mode of the ureide-chalcone hybrid structures CHA-233, CHA-236, CHA-383, CHA-384, CHA-392, and CHA-397 and interacting residues within the 3CLpro active site are shown in Figure 15B. As discussed earlier, these compounds were previously synthesized and reported as antimalarial agents [65]. The spatial orientation of these ureide-chalcones in the active site of the 3CLpro is precisely the same, and the ureide substructure formed two conventional hydrogen bonds by the interaction of Arg188 and the two NH atoms (Figure 15B).

**Figure 16:**
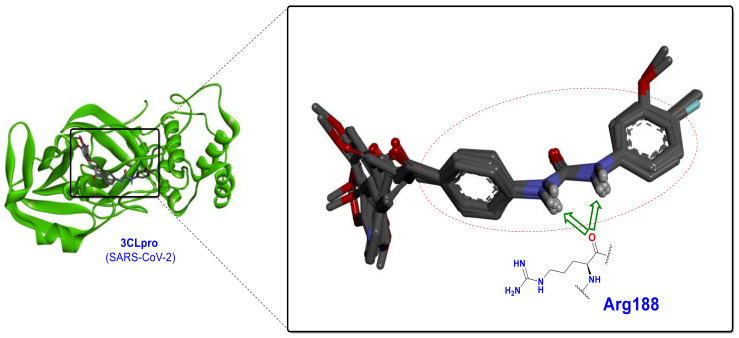
Spatial orientation and establishment of the two hydrogen bonds by the ureide-chalcone hybrid structures CHA-233, CHA-236, CHA-383, CHA-384, CHA-392, and CHA-397 with the carbonyl group of Arg188 at the active site of the SARS-CoV-2 3CLpro.

These hydrogen bonding interactions cause the urea to be oriented exactly at a specific location adjacent to the Arg188 (Figure 16). These ureide-chalcone hybrid structures have a high selectivity toward 3CLpro compared to PLpro and significantly produce stronger interactions than compound B (as standard) at the SARS-CoV-2 3CLpro active site.

### 3.4. Selective Ligands towards the SARS-CoV-2 PLpro

A long time after the emergence of the SARS-CoV pandemic in 2002, structure-based design approaches for the development of PLpro inhibitors were unsuccessful due to the insufficiency of the target structural information. For the first time in 2008, Ghosh and co-workers were able to identify two selective SARS-CoV PLpro inhibitors by performing an extensive high-throughput screening on 50,080 compounds [66]. Interestingly, GRL0617 (Figure 8A) with a chalcone-amide backbone is a well-known compound generated from the study of Ghosh et al., and, currently, it is used for the design and development of the SARS-CoV/SARS-CoV-2 PLpro inhibitors as a standard and lead compound. Herein, to validate the assessments and better understand the interactions, GRL0617 was used as a standard.

**Figure 17:**
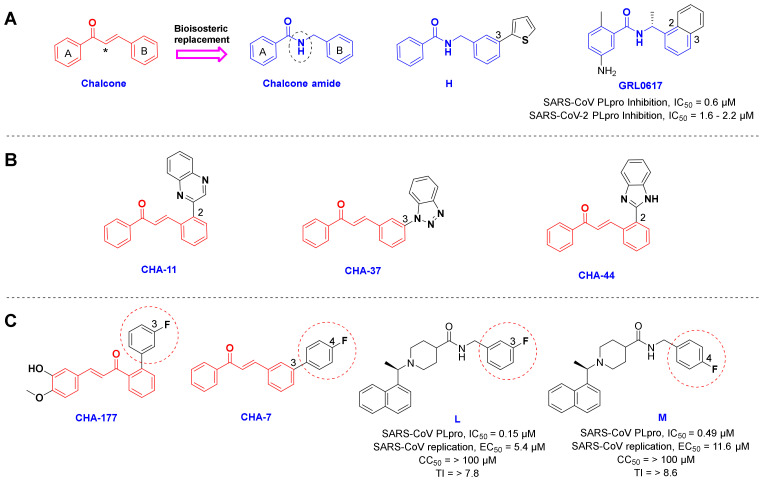
(**A**) Structural similarity of the chalcone and the chalcone amide scaffolds, and GRL0617 as a well-known inhibitor of SARS-CoV PLpro. (**B**) The structure of the predicted selective ligands CHA-11, CHA-37, and CHA-44 toward SARS-CoV-2 PLpro. (**C**) Structure of the fluorophenyl containing chalcones CHA-177 and CHA-7 and the previously reported compounds L and M. Asterisk in the structure of chalcone indicates the location of the bioisosteric replacement.

As mentioned in the results section, CHA-7, CHA-11, CHA-37, CHA-44, and CHA-177 showed superior docking scores among our chalcone-based compound library against the SARS-CoV-2 PLpro and host-based targets. Structural evaluation of the selective PLpro ligands shows outstanding similarities. In general, these selective PLpro compounds have unsubstituted and flat nitrogen-containing aromatic groups on ring B of the chalcone backbone (Figure 17A,B). Correspondingly, the quinoxaline ring in CHA-11, benzo-[1–3]triazole in CHA-37, and benzimidazole in CHA-44 in position 2 or 3 on the B-ring are the main motifs for binding. As indicated in the structure of GRL0617, the same position is occupied by a fused phenyl ring with the B-ring of the chalcone backbone. Interestingly, in a recent structure-based drug design attempt, extensive SAR evaluations have led to a chalcone-amide isoster bearing a 2-thienyl at position 3 of ring B for producing the strongest derivatives against SARS-CoV-2 PLpro (Figure 17A, structure H) [58]. The meta- and para-fluorophenyl substitutions in positions 2 and 3 of the B-ring also existed in the structure of CHA-7 and CHA-177, which were found to have high docking scores in our compound library. These substituents are also found in the structure of the highest affinity SARS-CoV PLpro inhibitors previously reported in the literature, which confirms and validates our results (Figure 17C, compounds L and M) [67]. Therefore, a fused and nitrogen-containing aromatic ring or a fluorophenyl ring substituted at position 3 of the chalcone B-ring (or chalcone-amide) is useful for increasing the inhibitory effect of compounds against the PLpro enzyme.

Tables S4 and S5 comparatively summarize all the predicted interactions related to the most promising CHA-12, CHA-37, CHA-378, and CHA-384 along with the appropriate standards baicalein and GRL0617 in the active site of the SARS-CoV-2 3CLpro and PLpro.

In addition to having a strong affinity of CHA-37 in the PLpro active site (rank 3, docking score = −9.5 kcal/mol), it is interesting that this ligand also creates remarkable interactions in the 3CLpro active site with the rank of 12 among the 757 chalcone-based compound library. Accordingly, we also examined the interactions of this compound in the active site of both the viral targets PLpro and 3CLpro.

### 3.5. Benzotriazole for Selective 3CLpro Inhibition

**Figure 18:**
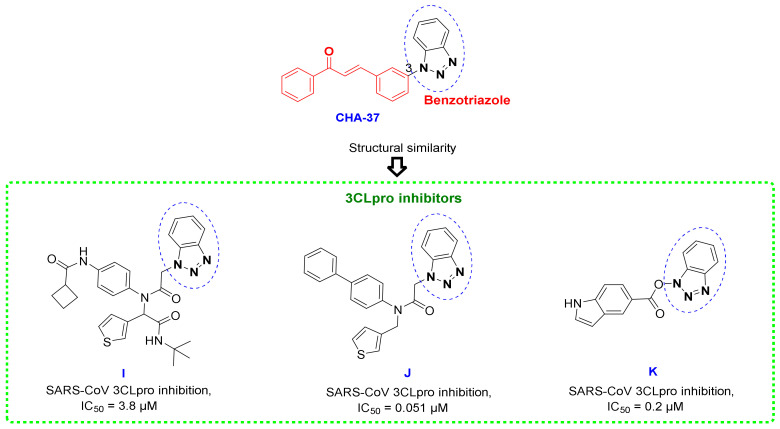
Chemical structures of CHA-37 and some potent SARS-CoV 3CLpro inhibitors containing benzotriazole scaffold.

One of the important reasons for creating strong interactions of CHA-37 in the 3CLpro active site could also be attributed to the presence of the benzotriazole group. This moiety has been observed in the structure of the previously reported and potent 3CLpro inhibitors, as existed in representative compounds I, J, and K (Figure 18) [68,69].

**Figure 19:**
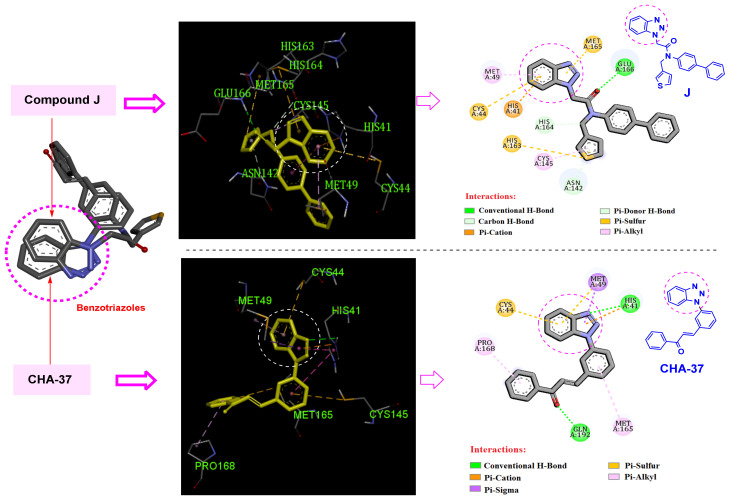
2D and 3D interactions of compound J as a highly potent reported SARS-CoV-2 3CLpro inhibitor and CHA-37 in the active site of the 3CLpro enzyme.

Comparison of the interactions of CHA-37 with compound J, a previously reported and highly potent SARS-CoV 3CLpro inhibitor (IC_50_ = 51 nM) [69], in the SARS-CoV-2 3CLpro active site shows that benzotriazole moieties of both compounds are occupying exactly the same space and mostly interact with the same residues His41, Cys44, and Met49 (Figure 19). The affinity of CHA-37 towards the 3CLpro binding space was further confirmed by the MM/PB(GB)SA calculations (Table 3).

## 4. Materials and Methods

### 4.1. Compound Library Preparation

PubChem database (accessed on 27 August 2020) was searched by keyword “Chalcone” and after applying Lipinski’s rule of 5, the number of 757 chalcone-based structures were retrieved, named CHA-1 to CHA-757, and used in this study (See the Supplementary Material for the mol2 format of our 757 compound library).

### 4.2. Target Selection and Preparation

Firstly, the structure of virus-based targets 3CLpro (PDB code: 6M2N) and PLpro (PDB code: 7JN2), and various host-based targets including p38 MAPK (PDB code: 4EH3), Cathepsin L (PDB code: 5MQY), CDK1 (PDB code: 6GU2), CDK2/CyclinA (PDB code: 6GUB), CDK9/cyclinT1 (PDB code: 3BLR), ERK2 (PDB code: 3SA0), HDAC2 (PDB code: 4LXZ), DHODH (PDB code: 5ZF7), CK2 alpha’ (PDB code: 5M4U), BRD2 (PDB code: 4J1P), BRD4 (PDB code: 6HOV), and sigma-1 receptor (PDB code: 6DK1), were extracted from the Protein Data Bank (www.rcsb.org) in PDB format. The preparation steps of proteins and ligands were performed based on the method described in our recent study [70].

The selected host-based and virus-based targets with the associated PDB codes as well as the basic parameters of the docking protocol are summarized in Table S6.

### 4.3. Virtual Screening

A Bash script in Linux was used for performing virtual screening by AutoDock Vina 1.1.2 (La Jolla, CA, USA) and ranking the bound conformations based on their best binding score of 757 chalcone-based compound library against the 14 selected virus and host-based targets. The center and size of the grid box in each target are presented in Table S6; the validation of the docking parameters was performed and the resultant conformation of the co-crystal re-docking operation is illustrated in Figure S2. The docking scores of all of the 757 chalcone structures within the 14 viral and host-based targets are in a Supplementary File (.xlsx file) [70].

Decoys selection in benchmarking datasets was performed by the Knime workflow Evotec_StructureBasedVirtualScreening_Calibration available on the Knime HUB https://hub.knime.com. Accordingly, 25 active compounds against 3CLpro and PLpro were collected from the literature and used in the Knime workflow to search a ZINC database of 7000 dissimilar compounds to find decoys that are similar to the active compounds by the molecular descriptors MW, nHBDs, nHBAs, logP, and nRTB but are topologically different in their Tanimoto similarity fingerprint. For benchmarking our structured based virtual screening method, 158 and 189 decoys were selected for 3CLpro and PLpro targets, respectively. Totally, 183 compounds for 3CLpro and 214 compounds for PLpro were used as a benchmarking dataset. Correspondingly, ROC curves and accuracy statistics were calculated by the relevant nodes in the Knime Analytics Platform 4.6.0 (Zurich, Switzerland).

### 4.4. Molecular Dynamics Simulation and MM/PB(GB)SA Calculations

GROMACS version 2020 installed on Ubuntu 20.04 Linux workstation was used for the dynamics simulation of compounds in the binding site in explicit water model TIP3P, and Amber99SB-ILDN force field was used for topology generation. The topology of ligands was generated by ACPYPE, and GAFF was used for ligand topology generation. Ligand atomic charges were calculated using PM3 in Gaussian 09 (Wallingford, CT, USA). The topology and coordinate files for the protein were generated using the pdb2gmx program of the GROMACS package, taking parameters from the Amber99SB-ILDN force field. The coordinate and topology files of the protein and the ligands were then merged to obtain the final starting structure and topology file for each complex.

The complex was centered in a dodecahedron periodic box and solvated by the addition of water molecules (simple point charge model). The total charge of the system was then neutralized by the addition of sodium and chloride ions as required. Sequentially, energy minimization was performed by the steepest descent algorithm. The system was then gradually heated to 300 K and was equilibrated for 100 ps using the NVT (constant volume and temperature) ensemble with position restraint for the heavy atoms followed by 100 ps equilibration in the NPT (constant pressure and temperature) ensemble at 1 atm. Both temperature and pressure were regulated using the Berendsen algorithm. Finally, the full system was subjected to 50 ns MD simulation with a 2 fs time step interval. The temperature and pressure were maintained at 300 K and 1 atm using the v-rescale temperature and Parrinello–Rahman pressure coupling method. The short-range non-bonded interactions were computed for the atom pairs within the cut-off of 1.2 nm, while the long-range electrostatic interactions were calculated using the Particle Mesh Ewald summation method with fourth-order cubic interpolation and 1.2 Å grid spacing. All h-bonds were constrained using the parallel LINCS method [71]. Finally, the Molecular Mechanics/Poisson Boltzmann (or Generalized Born) Surface Area (MM/PB(GB)SA) method for calculating the free energy of binding was accomplished using gmx_MMPBSA 1.6.0 introduced by Mario S. Valdés Tresanco [72]. gmx_MMPBSA is a new tool based on AMBER’s MMPBSA.py in AmberTools20 aiming to perform end-state free energy calculations with GROMACS files.

Molecular Mechanics/Poisson Boltzmann (or Generalized Born) Surface Area (MM/PB(GB)SA) is a post-processing method in which representative snapshots from an ensemble of conformations are used to calculate the free energy change between a bound and unbound state of a ligand in a receptor. Free energy differences are calculated by combining the so-called gas phase energy contributions (MM term) that are independent of the chosen solvent model as well as solvation-free energy components (electrostatic and hydrophobic) calculated from an implicit solvent model Poisson Boltzmann or Generalized Born for each species. Entropy contributions (−TΔS) to the total free energy were added using the so-called interaction entropy method, which is theoretically rigorous, computationally efficient, and numerically reliable for calculating the entropic contribution to free energy in protein–ligand binding and other interaction processes.

The gas phase free energy contributions were calculated using mmpbsa_py_energy (use_sander = 0) within the AmberTools package (San Francisco, CA, USA). The MM/PBSA equation was solved numerically using the pbsa program included with AmberTools20 (sander_apbs = 0). Hydrophobic contribution in MM/GBSA was approximated by the LCPO method (molsurf = 0) implemented within the sander and the total non-polar solvation-free energy in MM/PBSA was modeled as a single term linearly proportional to the solvent accessible surface area (inp = 1).

The following parameters were used for the calculation of binding energies using the MM/PB(GB)SA method. Start frame = 4900, end frame = 5000, interval = 20, entropy = interaction entropy, igb = 5, saltcon = 0.150, inp = 1, istrng = 0.150, fillratio = 4.0, and radiopt = 0.

The following formulas represent the calculation of the parameters and energy components:

ΔE = E_complex_ − E_receptor_ − E_ligand_

ΔG_gas_ = ΔG_VDW_ + ΔG_EEL_

For the MM/GBSA method: ΔG_solv_ = ΔG_EGB_ + ΔG_ESURF_

For the MM/PBSA method: ΔG_solv_ = ΔG_EPB_ + ΔG_ENPOLAR_

ΔG_total_ = ΔG_gas_ + ΔG_solv_

ΔG_b_ = ΔG_total_ + IE

### 4.5. Druglikeness and Pharmacokinetic Prediction

Lipinski’s rule of 5 and violations were calculated using PreADMET (https://preadmet.webservice.bmdrc.org), BBB permeation and GI absorption were estimated using SwissADME (http://www.swissadme.ch) and P-glycoprotein inhibition, and acute oral toxicity and carcinogenicity were calculated using admetSAR (http://lmmd.ecust.edu.cn/admetsar2) web-based servers.

## 5. Conclusions

Since 3CLpro and PLpro play a key role in the replication of SARS-CoV-2, these enzymes are the main therapeutic targets for the treatment of COVID-19. In addition, modulators of the host-based targets that have roles in the virus life cycle can assist in the suppression of SARS-CoV-2. In this study, a targeted and extensive computational evaluation was performed to investigate the affinity of 757 chalcone-based structures in the inhibition of the SARS-CoV-2 3CLpro and PLpro enzymes and modulation of a wide range of host-based targets. Our results indicated that the ureide substructure is an integral part of the potent and selective 3CLpro inhibition by hydrogen bonding to Arg188 and occupying the S1 subsite. Correspondingly, the sulfonamide moiety of CHA-378 can infer 3CLpro inhibition potential to some extent by occupying the S3 subsite and interacting with Gln189. CHA-384 as a high-affinity and selective 3CLpro inhibitor was introduced as a lead compound in our study to inhibit 3CLpro. A benzotriazole fragment was also found to be a well-interacting residue in the binding space of the 3CLpro and PLpro enzymes, which is evidenced by the introduction of CHA-37. These findings are in excellent accordance with the results of a recent large-scale fragment-based screening performed by mass spectrometry and X-ray crystallography. The established relaxant effects of CHA-12 on pulmonary smooth muscles through antagonistic activity on CysLT1, which was reported previously by in vivo and in vitro assessments, along with our predictions of its amazing effects on all virus and host-based targets promise the introduction of a valuable compound for the treatment of COVID-19.

Regarding the limitations of the study, perhaps the most important point is that it was not possible to experimentally test the capabilities of the identified compounds to inhibit SARS-CoV-2 and its PLpro and 3CLpro enzymes in vitro and in vivo. But what encourages us is that our obtained results have a high agreement with the previously reported experimental results, as well as the conclusive structure-activity relationships obtained for the SARS-CoV-2 PLpro/3CLpro inhibitors, which have been tried to be explained in detail. Therefore, we encourage researchers who have the facility to experimentally evaluate the potential of our introduced hits in inhibiting the replication of the virus and the enzymes 3CLpro/PLpro to act in this direction.

## Figures and Tables

**Table 1:** Overall accuracy metrics for the benchmarking datasets of 3CLPro and PLPro targets.

Target	Correct Classified	Wrong Classified	Accuracy	Error	Cohen’s Kappa (k)
3CLpro	147	36	80.32%	19.67%	0.40%
PLpro	173	41	80.84%	19.15%	0.38%

**Table 2:** Ranking of the most active hits identified by docking-based virtual screening against various virus and host-based targets. The selected HBATs are named based on their PDB codes. Chalcone-based compounds are named based on their number in our library and encoded as CHA-1 to CHA-757, and the numbers reported in the table body are their docking rank in each molecular target.

Chalcones	4EH3	5MQY	6GU2	6GUB	3BLR	3SA0	4LXZ	5ZF7	5M4U	4J1P	6HOV	6DK1	6M2N	7JN2
**CHA-7**	12	72	**5**	31	19	17	30	31	**6**	**6**	**6**	**4**	84	**5**
**CHA-10**	86	36	64	33	56	24	**1**	**4**	156	85	32	36	27	23
**CHA-11**	231	**5**	**10**	**1**	**2**	30	19	25	19	13	**2**	17	36	**6**
CHA-12 ^a^	**1**	**2**	**1**	**2**	**3**	**1**	**2**	**1**	**2**	**2**	**3**	**1**	**1**	**1**
**CHA-15**	17	**6**	**6**	**8**	39	24	20	**10**	11	65	52	**8**	**10**	118
**CHA-22**	153	337	109	263	312	61	566	37	**4**	191	340	48	161	69
**CHA-29**	157	220	34	23	25	**5**	266	145	60	**3**	43	236	29	99
**CHA-30**	159	42	53	123	49	33	**3**	39	209	69	422	52	30	321
**CHA-34**	**4**	29	242	96	107	108	14	28	61	50	25	109	52	35
CHA-37	**2**	**4**	**3**	**9**	**7**	**4**	34	**9**	**1**	**5**	**1**	**2**	12	**3**
**CHA-44**	662	16	**8**	**3**	**1**	28	**10**	73	118	43	**5**	53	75	**4**
**CHA-60**	**5**	69	358	65	36	49	448	250	123	631	382	44	187	534
**CHA-77**	13	206	**9**	**4**	37	**8**	136	653	463	63	14	226	193	298
**CHA-84**	**3**	650	29	256	753	431	74	756	755	594	716	757	399	755
**CHA-86**	190	582	381	307	191	214	310	259	79	84	62	62	114	**7**
**CHA-118**	674	684	443	529	503	305	654	315	703	439	672	737	405	**8**
**CHA-134**	87	384	65	96	107	53	**5**	556	685	**7**	23	228	52	44
**CHA-166**	410	291	30	19	20	26	78	142	54	**4**	35	159	90	615
**CHA-177**	31	39	31	42	**6**	45	**9**	48	**7**	**8**	**9**	55	91	**9**
**CHA-178**	411	553	623	160	199	223	**4**	67	107	373	133	128	116	240
**CHA-206**	18	212	228	21	85	**3**	79	143	206	**9**	78	78	159	13
**CHA-215**	357	**7**	134	11	58	54	59	**3**	85	115	16	15	611	567
**CHA-233**	20	27	39	29	130	17	233	20	43	117	101	12	**8**	401
**CHA-234**	197	**3**	32	22	159	19	146	13	22	37	283	**5**	40	96
**CHA-236**	**7**	**1**	14	13	251	13	114	**7**	36	118	79	**6**	**3**	249
**CHA-282**	49	122	309	164	73	71	82	108	58	28	226	107	23	**2**
**CHA-297**	158	167	15	**6**	**10**	14	63	16	59	**10**	18	37	11	27
**CHA-298**	50	56	33	45	15	21	24	21	86	38	10	9	68	34
**CHA-375**	**9**	86	41	128	**8**	15	26	**5**	62	30	11	18	32	19
CHA-378	**10**	**10**	24	**5**	**4**	16	15	22	63	**1**	**4**	28	**4**	**10**
**CHA-383**	373	344	117	393	660	**7**	513	55	574	515	430	29	**6**	663
CHA-384	11	88	**7**	47	63	**2**	158	**2**	25	72	**7**	**3**	**2**	37
**CHA-392**	74	89	55	58	51	34	579	14	261	73	**8**	14	**5**	505
**CHA-397**	24	90	43	48	43	**5**	126	12	90	102	12	**10**	**7**	260
**CHA-485**	99	288	**4**	15	34	142	87	388	119	265	300	206	**9**	59
**CHA-509**	175	693	18	61	5	48	39	15	176	16	251	59	79	60
**CHA-567**	81	12	59	63	77	121	170	51	**5**	20	26	60	44	64
**CHA-581**	41	443	99	140	44	49	69	30	**3**	57	27	**7**	233	64
**CHA-734**	43	204	**2**	40	95	96	56	700	462	12	13	443	111	384

^a^: Bolded ligands are the best hits selected based on their rank number for further evaluations. 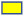 Most active ligands selective towards the PLpro target. Lighter colors indicate a lower magnitude of affinity. 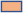 Most active ligands towards 3CLpro and PLpro targets. Lighter colors indicate a lower magnitude of affinity. 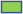 Most active ligands selective towards the 3CLpro target. Lighter colors indicate a lower magnitude of affinity. Lighter colors of each one represent lower significance of the compound.

**Table 3:** Binding free energy values and energy components of the compounds in the 3CLpro active site calculated by MM/PB(GB)SA in kcal/mol.

Compounds	MM/GBSA				MM/PBSA				
	ΔG_b_	ΔG_Total_	ΔG_solv_	ΔG_gas_	ΔG_b_	ΔG_Total_	ΔG_solv_	ΔG_gas_	IE
Baicalein	−29.43	−28.77	5.51	−34.28	−24.23	−23.57	10.71	−34.28	0.04
CHA-12	−44.03	−47.62	20.65	−68.26	−35.57	−39.16	29.11	−68.26	0.04
CHA-384	−40.05	−42.32	15.44	−57.76	−30.39	−32.65	25.10	−57.76	0.04
CHA-37	−25.05	−27.17	13.54	−40.70	−17.80	−19.91	20.79	−40.70	0.04
CHA-297	−14.80	−21.44	26.11	−47.55	−14.53	−21.17	26.38	−47.55	0.04
CHA-378	−24.98	−26.83	27.92	−54.75	−24.28	−26.13	28.62	−54.75	0.04
Hirsutenone	−29.37	−29.28	9.42	−38.70	−20.66	−20.58	18.12	−38.70	0.1
A	−9.43	−11.18	19.86	−31.05	−14.21	−15.96	15.08	−31.05	0.03
B	−19.41	−20.85	8.6	−29.45	−13.22	−14.66	14.79	−29.45	1.53

**Table 4:** Binding free energy values and energy components of the compounds in the PLpro active site calculated by MM/PB(GB)SA in kcal/mol.

Compounds	MM/GBSA				MM/PBSA				
	ΔG_b_	ΔG_T_	ΔG_solv_	ΔG_gas_	ΔG_b_	ΔG_T_	ΔG_solv_	ΔG_gas_	IE
Co-ligand ^a^	−12.36	−13.65	7.67	−21.32	−11.99	−13.28	8.04	−21.32	0.04
GRL0617	−34.65	−36.42	19.37	−55.80	−26.64	−28.41	27.39	−55.80	0.03
CHA−12	−35.81	−35.60	14.20	−49.81	−31.13	−30.92	18.89	−49.81	0.16
CHA-37	−21.23	−20.35	6.83	−27.18	−14.11	−13.22	13.95	−27.18	0.04
CHA-378	−20.52	−27.87	39.46	−67.33	−16.31	−23.66	43.68	−67.33	0.04
Hirsutenone	−23.11	−23.53	13.47	−37.00	−19.61	−20.03	16.97	−37.00	0.93

^a^: Co-crystal ligand.

## Data Availability

Not applicable.

## Supplementary Materials

The following supporting information can be downloaded at: https://www.mdpi.com/article/10.3390/ijms24108789/s1.
