# Supplementary material for: Discovery of Chalcone-Based Hybrid Structures as High Affinity and Site-Specific Inhibitors against SARS-CoV-2: A Comprehensive Structural Analysis Based on Various Host-Based and Viral Targets
Source: Int J Mol Sci. 2023 May 15;24(10):8789. doi: 10.3390/ijms24108789 (PMC10218655; doi:10.3390/ijms24108789)
Supplement: Supplementary file 1 [file ijms-24-08789-s001.zip › ijms-2133167-Supplementary materials.pdf]

International Journal of
*Molecular Sciences*

MDPI

*Supplementary Materials*

# Discovery of Chalcone-Based Hybrid Structures as High Affinity and Site-Specific Inhibitors against SARS-CoV-2: A Comprehensive Structural Analysis Based on Various Host-Based and Viral Targets

**Mehdi Valipour [1], Silvia Di Giacomo [2], Antonella Di Sotto [2,*], Hamid Irannejad [3,*]**

[1] Razi Drug Research Center, Iran University of Medical Sciences, Tehran P9XH+4QJ, Iran
[2] Department of Physiology and Pharmacology "V. Erspamer", Sapienza University of Rome, P.le Aldo Moro 5, 00185 Rome, Italy
[3] Department of Medicinal chemistry, Faculty of Pharmacy, Mazandaran University of Medical Sciences, Sari H27P+84G, Iran
* Correspondence: antonella.disotto@uniroma1.it (A.D.S.); irannejadhamid@gmail.com (H.I.); Tel.: +98-9124572673 (H.I.)

**Citation:** Valipour, M.; Di Giacomo, S.; Di Sotto, A.; Irannejad, H. Discovery of Chalcone-based Hybrid Structures as High Affinity and Site-Specific Inhibitors against SARS-CoV-2: A Comprehensive Structural Analysis based on Various Host-based and Viral Targets. *Int. J. Mol. Sci.* **2022**, *23*, x. https://doi.org/10.3390/xxxxx

Academic Editors: Adam Jarmuła and Piotr Maj

Received: 15 December 2022
Revised: 1 May 2023
Accepted: 8 May 2023
Published: 15 May 2023

**Copyright:** © 2022 by the authors. Submitted for possible open access publication under the terms and conditions of the Creative Commons Attribution (CC BY) license (https://creativecommons.org/licenses/by/4.0/).

**Table S1.** The most important host and virus-based targets selected to evaluate the antiviral activity of the 757 chalcone-based compound library, along with PDB codes, chemical structure of their co-crystal ligands, and the authoritative studies proposing these targets as promising strategies for the treatment of viral diseases such as COVID-19.

| N. | HBATs | PDB Code | Co-crystallized ligand | HBATs-References |
|---|---|---|---|---|
| **1** | p38 MAPK | 4EH3 | 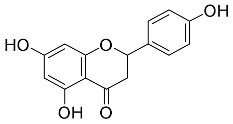 Naringenin | [1-8] |
| **2** | Cathepsin L | 5MQY | 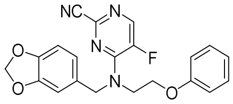 PubChem CID: 137653100 | [9-16] |
| **3** | CDK1 | 6GU2 | 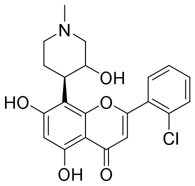 Flavopiridol | [17-22] |
| **4** | CDK2/CyclinA | 6GUB | 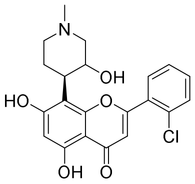 Flavopiridol | [23-25] |
| **5** | CDK9/cyclinT1 | 3BLR | 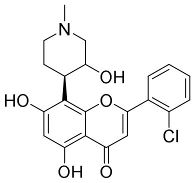 Flavopiridol | [26-29] |
| **6** | ERK2 | 3SA0 | 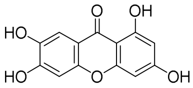 Norathyriol | [30-34] |
| **7** | HDAC2 | 4LXZ | 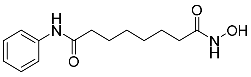 Vorinostat | [35-39] |
| **8** | DHODH | 5ZF7 | 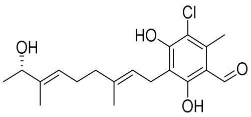 3vva | [40-46] |
| **9** | CK2 alpha' | 5M4U | 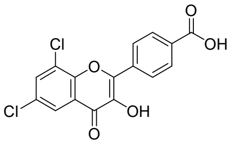 PubChem CID: 38997316 | [47-52] |
| **10** | BRD2 | 4J1P | 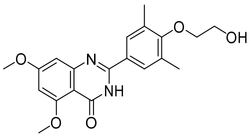 Apabetalone | [53-55] |

| | | | | |
|---|---|---|---|---|
| **11** | BRD4 | 6HOV | 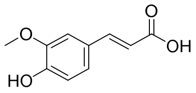 Ferulic acid | [53, 55-57] |
| **12** | Sigma-1 receptor | 6DK1 | 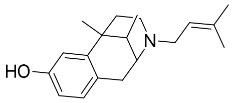 Pentazocine | [58-64] |
| **13** | 3CLpro | 6M2N | 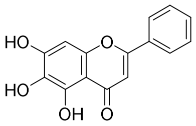 Baicalein | [65-68] |
| **14** | PLpro | 7JN2 | 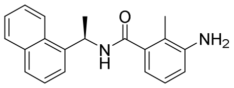 PubChem CID: 153835436 | [69-72] |

**Table S2.** Some important druglikeness, ADME, and toxicity parameters of the best identified **CHA-12**, **CHA-37**, **CHA-378**, **CHA-384**, and standards quercetin and resveratrol predicted by admetSAR, preADME, and swissADME online servers.

| **Parameters** | **CHA-12** | **CHA-37** | **CHA-378** | **CHA-384** | **Quercetin** | **Resveratrol** |
|---|---|---|---|---|---|---|
| **Formula** | $C_{26}H_{19}N_5O_3$ | $C_{21}H_{15}N_3O$ | $C_{22}H_{19}NO_5S$ | $C_{23}H_{17}ClN_2O_4$ | $C_{15}H_{10}O_7$ | $C_{14}H_{12}O_3$ |
| **Molecular weight (g/mol)** | 449.46 | 325.36 | 409.45 | 420.85 | 302.24 | 228.24 |
| **Lipinski rule of Five** | Suitable | Suitable | Suitable | Suitable | Suitable | Suitable |
| **Rule of Five Violations a** | 0 | 0 | 0 | 0 | 0 | 0 |
| **BBB permeant** | No | Yes | No | No | No | Yes |
| **GI absorption** | High | High | High | High | High | High |
| **P-glycoprotein Inhibitor** | No | Inhibitor | No | No | No | No |
| **Acute Oral Toxicity** | III | III | III | III | II | III |
| **Carcinogens** | No | No | No | No | No | No |

a: Violations of Lipinski rule of 5 (logP < 5, MW < 500, nHBA < 10, and nHBD < 5).

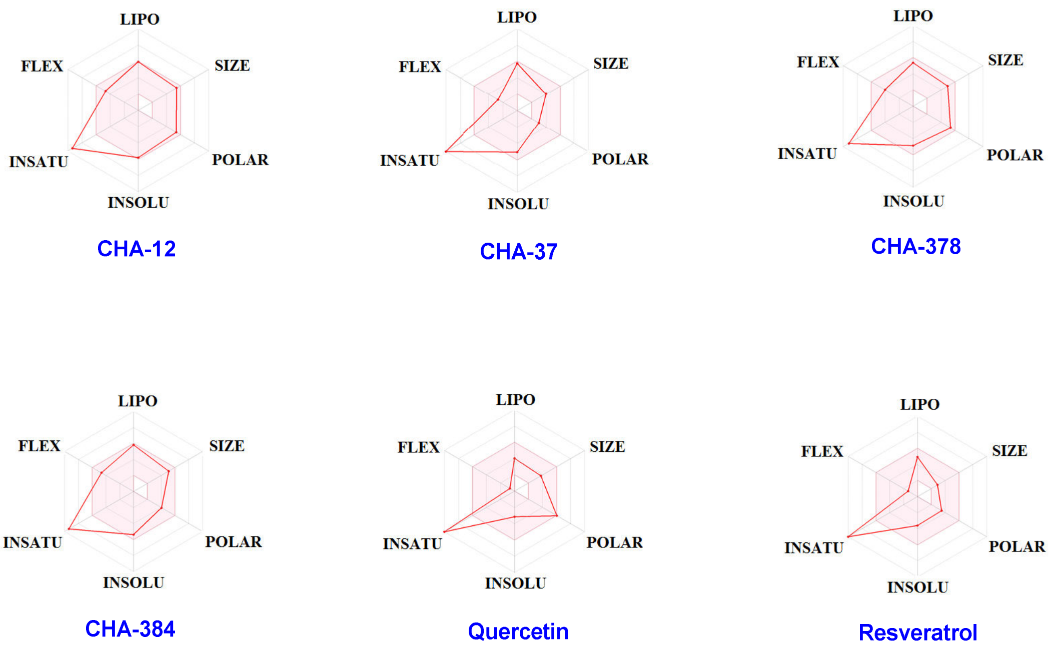


**Figure S1.** Radar map of the physicochemical properties for oral bioavailability of **CHA-12**, **CHA-37**, **CHA-378**, **CHA-384**, and standards quercetin and resveratrol predicted by SwissADME online server (colored zone is the suitable physicochemical space for oral bioavailability). LIPO (Lipophilicity): -0.7 < XLOGP3 < +5.0**;** SIZE: 150 g/mol < mw < 500 g/mol**;** POLAR (polarity): 20 Å$^2$ < TPSA < 130 Å$^2$**;** INSOLU (insolubility): 0 < Log S (ESOL) < 6**;** INSATU (insaturation): 0.25 < Fraction Csp3 < 1**;** FLEX (Flexibility): 0 < Numb. rotatable bonds < 9.

**Table S3.** Predicted inhibitory effects of **CHA-12**, **CHA-37**, **CHA-378**, **CHA-384**, resveratrol, and quercetin on cytochrome P450 enzymes (predicted by preADME online server).

| **Compounds** | **CYP2C19 inhibition** | **CYP2C9 inhibition** | **CYP2D6 inhibition** | **CYP2D6 Substrate** | **CYP3A4 inhibition** | **CYP3A4 Substrate** |
|---|---|---|---|---|---|---|
| **CHA-12** | Non | Inhibitor | Non | Non | Non | Weakly |
| **CHA-37** | Non | Inhibitor | Non | Non | Non | Weakly |
| **CHA-378** | Inhibitor | Inhibitor | Non | Non | Inhibitor | Non |
| **CHA-384** | Non | Inhibitor | Non | Non | Non | Non |
| **Resveratrol** | Inhibitor | Inhibitor | Non | Non | Inhibitor | Non |
| **Quercetin** | Inhibitor | Inhibitor | Non | Non | Inhibitor | Non |

**Table S4.** Important interactions of most active **CHA-12**, **CHA-378**, **CHA-384,** and baicalein as standard in the SARS-CoV-2 3CLpro active site.

| **Interactions** | **CHA-12** | | **CHA-378** | | **CHA-384** | | **Baicalein** | |
|---|---|---|---|---|---|---|---|---|
| | Residues | Interacting groups | Residues | Interacting groups | Residues | Interacting groups | Residues | Interacting groups |
| **Conventional H-bond** | Glu166 | NH-Tetrazole | Asn142 | 3-OH (B-Phenyl ring) | Arg188 | 1-NH (Urea-moiety) | Gly143 | 6-OH (A-Ring) |
| | Thr190 | OH (A-Phenyl ring) | | | Arg188 | 2-NH (Urea-moiety) | Ser144 | 6-OH (A-Ring) |
| | Gln192 | Carbonyl group | | | | | | |
| | His41 | O (Etheric linker) | | | | | | |
| | His41 | N-Quinoline | | | | | | |
| **Pi-donor** | Gln189 | B-Phenyl ring | Asn142 | B-Phenyl ring | | | | |
| **Pi-Alkyl** | Met49 | B-Phenyl ring | Met49 | Phenyl ring (Sulf-moiety) | Met49 | A-Phenyl ring | Met49 | B-Ring |
| | Cys145 | Quinoline (Benzene ring) | Met165 | A-Phenyl ring | Met165 | A-Phenyl ring | Cys44 | B-Ring |
| | | | Cys145 | B-Phenyl ring | Pro168 | Phenyl ring (Urea- | Cys145 | C-Ring |

| | | | | | | | | |
|---|---|---|---|---|---|---|---|---|
| | | | | | | moiety) | | |
| | | | His41 | Methyl (Sulf-moiety) | | | | |
| **Pi-Sulfur** | Met49 | Quinoline (Pyridine ring) | | | | | Cys145 | A-Ring |
| **Pi-Sigma** | Thr25 | Quinoline (Pyridine ring) | Gln189 | A-Phenyl ring | Thr25 | B-Phenyl ring | | |
| | | | Gln189 | A-Phenyl ring | | | | |
| **Acceptor-Acceptor** | | | Asn142 | 4-OH (B-Phenyl ring) | | | | |
| | | | Ser144 | 4-OH (B-Phenyl ring) | | | | |
| **Donor-Donor** | | | | | | | Gly143 | 5-OH (A-Ring) |
| **Pi-Cation** | | | His41 | Phenyl ring (Sulf-moiety) | | | | |
| **Pi-Pi Stacked** | | | His41 | Phenyl ring (Sulf-moiety) | His41 | A-Phenyl ring | His41 | B-Ring |
| **Alkyl** | | | | | Pro168 | Cl (Urea-moiety) | | |
| | | | | | Ala191 | Cl (Urea-moiety) | | |
| **Carbon** | | | | | | | Met165 | Carbonyl (C-Ring) |

**Table S5.** Important interactions of the most active **CHA-12**, **CHA-37**, **CHA-378** and standard compound GRL0617 in the active site of SARS-CoV-2 PLpro.

| **Interactions** | **CHA-12** | | **CHA-37** | | **CHA-384** | | **GRL0617** | |
|---|---|---|---|---|---|---|---|---|
| | Residues | Interacting groups | Residues | Interacting groups | Residues | Interacting groups | Residues | Interacting groups |
| **Conventional H-bond** | Lys157 | N1 Tetrazole | | | Tyr264 | Carbonyl group | Gln269 | Carbonyl group |
| | Gln269 | N4 Tetrazole | | | Glu167 | NH (Sulf-moiety) | Asp164 | NH (Amide) |
| | Tyr264 | OH (A-Phenyl ring) | | | | | Gly163 | NH2 (A-Phenyl ring) |
| **Pi-donor** | Gln269 | Tetrazole ring | | | | | | |
| **Pi-Alkyl** | Pro248 | B-Phenyl ring | Pro248 | B-Phenyl ring | Pro248 | B-Phenyl ring | Tyr268 | $CH_3$ (A-Phenyl ring) |
| | | | Pro248 | Triazole (Benzotriazole) | Leu162 | A-Phenyl ring | Pro247 | Naphthyl (Closer ring) |
| | | | Pro248 | Benzene (Benzotriazole) | | | Pro248 | Naphthyl (Closer ring) |
| | | | Pro247 | Benzene (Benzotriazole) | | | Pro248 | Naphthyl (Farther ring) |
| **Amide-Pi Stacked** | Gly163 | A-Phenyl ring | Gly163 | A-Phenyl ring | Gly163 | A-Phenyl ring | | |
| | | | Asn267 | Triazole (Benzotriazole) | | | | |
| **Pi-Sigma** | | | | | Gln269 | A-Phenyl ring | Asp164 | A-Phenyl ring |
| **Acceptor-Acceptor** | Tyr273 | Carbonyl group | | | | | | |
| **Pi-Cation** | | | Lys157 | A-Phenyl ring | | | | |
| **Pi-Anion** | Glu167 | Tetrazole ring | | | Asp164 | B-Phenyl ring | Asp164 | A-Phenyl ring |
| | | | | | Glu167 | Phenyl ring (Sulf-moiety) | | |

| | | | | | | | | |
|---|---|---|---|---|---|---|---|---|
| **Pi-Pi Stacked** | Tyr268 | Quinoline (Pyridine ring) | Tyr264 | B-Phenyl ring | | | | |
| | Tyr268 | Quinoline (Benzene ring) | | | | | | |
| **Pi-Pi T-Shaped** | Tyr268 | B-Phenyl ring | Tyr268 | Benzene (Benzotriazole) | Tyr264 | B-Phenyl ring | Tyr268 | Naphthyl (Closer ring) |
| | | | | | | | Tyr268 | Naphthyl (Farther ring) |
| **Carbon** | | | | | Pro248 | 4-OH (B-Phenyl ring) | | |

**Table S6.** The list of host-based and virus-based targets along with their associated PDB codes as well as the essential parameters for the docking protocol used in this study.

| Entry | Target | PDB Code | X | Y | Z | Grid box | Docking score[a] |
|---|---|---|---|---|---|---|---|
| **1** | p38 MAPK | 4EH3 | -2.668 | 0.007 | -20836 | 20,20,20 | - 8.40 |
| **2** | Cathepsin L | 5MQY | 54.336 | 48.381 | 17.63 | 20,20,20 | -6.80 |
| **3** | CDK1 | 6GU2 | 329.218 | 212.541 | 192.325 | 20,20,20 | -9.60 |
| **4** | CDK2/CyclinA | 6GUB | -8.411 | -21.607 | 22.226 | 20,20,20 | -10.10 |
| **5** | CDK9/cyclinT1 | 3BLR | 52.813 | -16.066 | -12.998 | 20,20,20 | -10.30 |
| **6** | ERK2 | 3SA0 | -12.826 | 10.831 | 40.627 | 26,22,20 | -7.40 |
| **7** | HDAC2 | 4LXZ | 25.00 | -17.005 | -0.024 | 20,20,20 | -7.50 |
| **8** | DHODH | 5ZF7 | -33.443 | 14.428 | -21.466 | 20,22.22 | -9.60 |
| **9** | CK2 alpha' | 5M4U | 10.536 | -19.326 | -8.976 | 20,20,20 | -11.40 |
| **10** | RBD2 | 4J1P | 10.105 | 20.989 | -6.705 | 20,20,26 | -7.30 |
| **11** | RBD4 | 6HOV | 10.887 | 5.487 | -0.524 | 20,20,20 | -6.50 |
| **12** | Sigma-1 receptor | 6DK1 | 12.009 | 37.935 | -33.62 | 20,20,22 | -9.0 |
| **13** | SARS-CoV-2 3CLpro | 6M2N | -32.488 | -63.85 | 40.962 | 22,20,20 | -7.80 |
| **14** | SARS-CoV-2 PLpro | 7JN2 | 51.608 | 30.592 | 0.79 | 20,20,20 | -8.80 |

a: Docking score of the re-dock co-crystal ligand for the method validation.

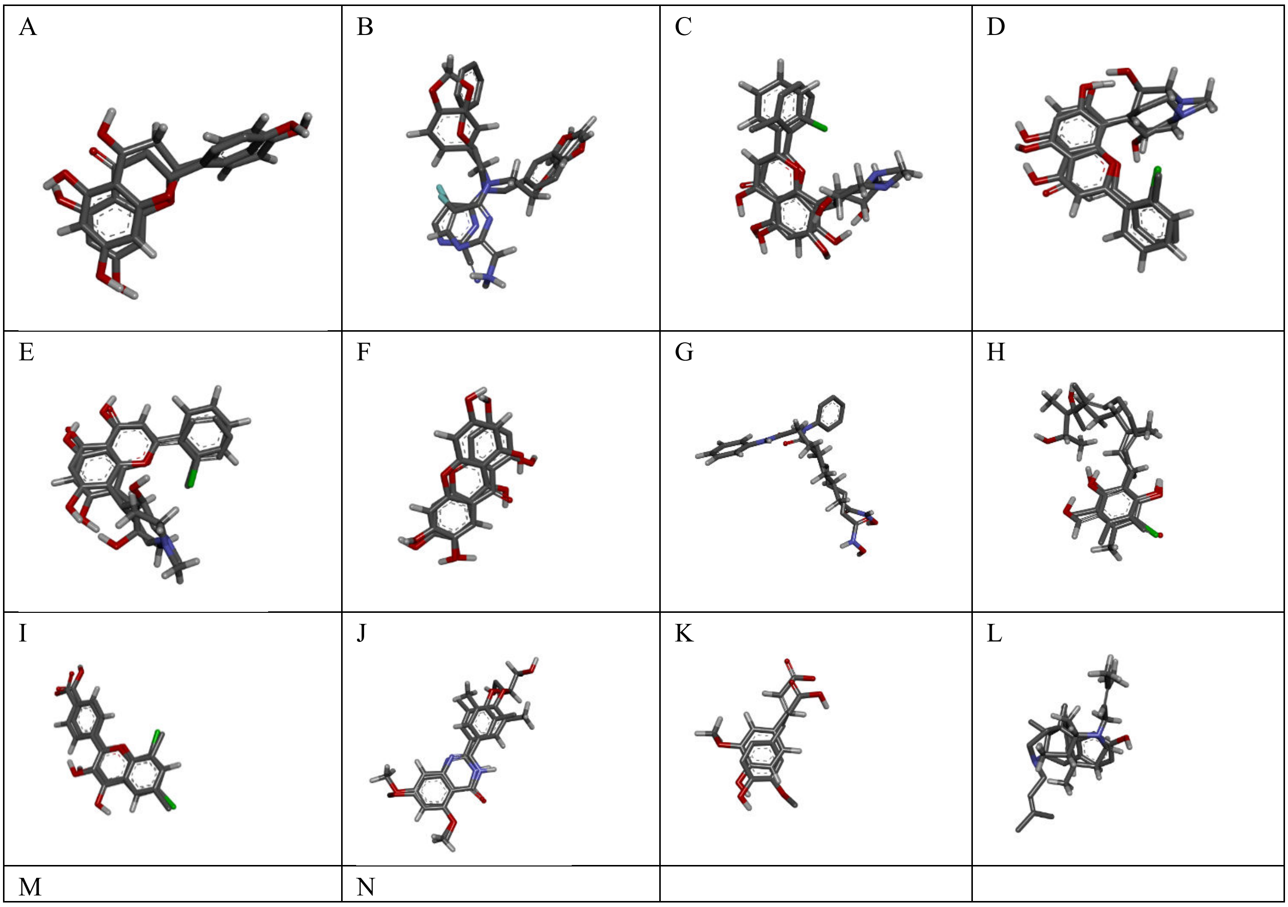

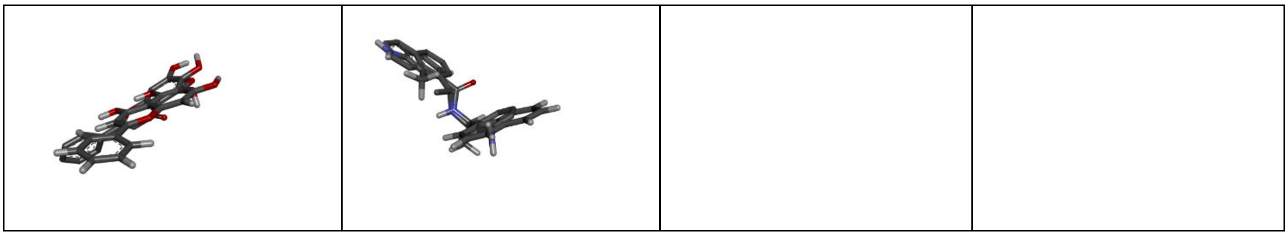

**Figure S2.** Coordinates of the co-crystal ligand and the re-docked one in the validation of the docking methods with (**A**) p38 MAPK, (**B**) Cathepsin L., (**C**) cyclin-dependent Kinase-1, (**D**) CDK2/CyclinA, (**E**) CDK9/cyclinT1, (**F**) ERK2, (**G**) HDAC2, (**H**) DHODH, (**I**) CK2 alpha, (**J**) RBD2, (**K**) RBD4, (**L**) sigma-1 receptor, (**M**) 3CLpro and (**N**) PLpro.

## References


1. Kopecky-Bromberg, S. A.; Martinez-Sobrido, L.; Palese, P., 7a protein of severe acute respiratory syndrome coronavirus inhibits cellular protein synthesis and activates p38 mitogen-activated protein kinase. *J. Virol.* **2006,** *80,* 785-793.
2. Marchant, D.; Singhera, G. K.; Utokaparch, S.; Hackett, T. L.; Boyd, J. H.; Luo, Z.; Si, X.; Dorscheid, D. R.; McManus, B. M.; Hegele, R. G., Toll-like receptor 4-mediated activation of p38 mitogen-activated protein kinase is a determinant of respiratory virus entry and tropism. *J. Virol.* **2010,** *84,* 11359-11373.
3. Mizutani, T.; Fukushi, S.; Saijo, M.; Kurane, I.; Morikawa, S., Phosphorylation of p38 MAPK and its downstream targets in SARS coronavirus-infected cells. *Biochem. Biophys. Res. Commun.* **2004,** *319*, 1228-1234.
4. Grimes, J. M.; Grimes, K. V., p38 MAPK inhibition: A promising therapeutic approach for COVID-19. *J. Mol. Cell. Cardiol.* **2020,** *144*, 63-65.
5. Kono, M.; Tatsumi, K.; Imai, A. M.; Saito, K.; Kuriyama, T.; Shirasawa, H., Inhibition of human coronavirus 229E infection in human epithelial lung cells (L132) by chloroquine: involvement of p38 MAPK and ERK. *Antivir. Res.* **2008,** *77*, 150-152.
6. Banerjee, S.; Narayanan, K.; Mizutani, T.; Makino, S., Murine coronavirus replication-induced p38 mitogen-activated protein kinase activation promotes interleukin-6 production and virus replication in cultured cells. *J. Virol.* **2002,** *76*, 5937-5948.
7. Padhan, K.; Minakshi, R.; Towheed, M. A. B.; Jameel, S., Severe acute respiratory syndrome coronavirus 3a protein activates the mitochondrial death pathway through p38 MAP kinase activation. *J. Gen. Virol.* **2008,** *89*, (8), 1960-1969.
8. McCaskill, J. L.; Ressel, S.; Alber, A.; Redford, J.; Power, U. F.; Schwarze, J.; Dutia, B. M.; Buck, A. H., Broad-spectrum inhibition of respiratory virus infection by microRNA mimics targeting p38 MAPK signaling. *Mol. Ther. -Nucleic Acids* **2017,** *7*, 256-266.
9. Liu, T.; Luo, S.; Libby, P.; Shi, G.-P., Cathepsin L-selective inhibitors: A potentially promising treatment for COVID-19 patients. *Pharmacol. Ther.* **2020**, 107587.
10. Huang, I.-C.; Bosch, B. J.; Li, F.; Li, W.; Lee, K. H.; Ghiran, S.; Vasilieva, N.; Dermody, T. S.; Harrison, S. C.; Dormitzer, P. R., SARS coronavirus, but not human coronavirus NL63, utilizes cathepsin L to infect ACE2-expressing cells. *J. Biol. Chem.* **2006,** *281*, 3198-3203.
11. Simmons, G.; Gosalia, D. N.; Rennekamp, A. J.; Reeves, J. D.; Diamond, S. L.; Bates, P., Inhibitors of cathepsin L prevent severe acute respiratory syndrome coronavirus entry. *Proc. Natl. Acad. Sci.* **2005,** *102*, 11876-11881.
12. Zhao, M.-M.; Yang, W.-L.; Yang, F.-Y.; Zhang, L.; Huang, W.-J.; Hou, W.; Fan, C.-F.; Jin, R.-H.; Feng, Y.-M.; Wang, Y.-C., Cathepsin L plays a key role in SARS-CoV-2 infection in humans and humanized mice and is a promising target for new drug development. *Signal Transduct. Target. Ther.* **2021,** *6*, 1-12.

13. Sacco, M. D.; Ma, C.; Lagarias, P.; Gao, A.; Townsend, J. A.; Meng, X.; Dube, P.; Zhang, X.; Hu, Y.; Kitamura, N., Structure and inhibition of the SARS-CoV-2 main protease reveal strategy for developing dual inhibitors against Mpro and cathepsin L. *Sci. Adv.* **2020,** *6*, eabe0751.
14. Bosch, B. J.; Bartelink, W.; Rottier, P. J., Cathepsin L functionally cleaves the severe acute respiratory syndrome coronavirus class I fusion protein upstream of rather than adjacent to the fusion peptide. *J. Virol.* **2008,** *82*, 8887-8890.
15. Ferraro, F.; Merlino, A.; dell′ Oca, N.; Gil, J.; Tort, J. F.; Gonzalez, M.; Cerecetto, H.; Cabrera, M.; Corvo, I., Identification of chalcones as Fasciola hepatica cathepsin L inhibitors using a comprehensive experimental and computational approach. *PLoS Negl. Trop. Dis.* **2016,** *10*, e0004834.
16. Garg, S.; Raghav, N., Inhibitory potential of some chalcones on cathepsins B, H and L. *RSC Adv.* **2015,** *5*, 72937-72949.
17. Surjit, M.; Liu, B.; Chow, V. T.; Lal, S. K., The nucleocapsid protein of severe acute respiratory syndrome-coronavirus inhibits the activity of cyclin-cyclin-dependent kinase complex and blocks S phase progression in mammalian cells. *J. Biol. Chem.* **2006,** *281*, 10669-10681.
18. Gutierrez-Chamorro, L.; Felip, E.; Ezeonwumelu, I. J.; Margelí, M.; Ballana, E., Cyclin-dependent Kinases as Emerging Targets for Developing Novel Antiviral Therapeutics. *Trends Microbiol.* **2021**.
19. Schang, L. M., Cyclin-dependent kinases as cellular targets for antiviral drugs. *J. Antimicrob. Chemother.* **2002,** *50*, 779-792.
20. Gargouri, M.; Alzwi, A.; Abobaker, A., Cyclin dependent kinase inhibitors as a new potential therapeutic option in management of COVID-19. *Med. Hypotheses* **2021,** *146*, 110380.
21. Heaton, B. E.; Trimarco, J. D.; Hamele, C. E.; Harding, A. T.; Tata, A.; Zhu, X.; Tata, P. R.; Smith, C. M.; Heaton, N. S., SRSF protein kinases 1 and 2 are essential host factors for human coronaviruses including SARS-CoV-2. *BioRxiv* **2020**.
22. Cribier, A.; Descours, B.; Valadão, A. L. C.; Laguette, N.; Benkirane, M., Phosphorylation of SAMHD1 by cyclin A2/CDK1 regulates its restriction activity toward HIV-1. *Cell Rep.* **2013,** *3*, 1036-1043.
23. Ludgate, L.; Ning, X.; Nguyen, D. H.; Adams, C.; Mentzer, L.; Hu, J., Cyclin-dependent kinase 2 phosphorylates s/tp sites in the hepadnavirus core protein C-terminal domain and is incorporated into viral capsids. *J. Virol.* **2012,** *86*, 12237-12250.
24. He, W.; Staples, D.; Smith, C.; Fisher, C., Direct activation of cyclin-dependent kinase 2 by human papillomavirus E7. *J. Virol.* **2003,** *77*, 10566-10574.
25. Bouchard, M.; Giannakopoulos, S.; Wang, E. H.; Tanese, N.; Schneider, R. J., Hepatitis B virus HBx protein activation of cyclin A–cyclin-dependent kinase 2 complexes and G1 transit via a Src kinase pathway. *J. Virol.* **2001,** *75*, 4247-4257.
26. Wang, S.; Fischer, P. M., Cyclin-dependent kinase 9: a key transcriptional regulator and potential drug target in oncology, virology and cardiology. *Trends Pharmacol. Sci.* **2008,** *29*, 302-313.
27. Khan, S. Z.; Mitra, D., Cyclin K inhibits HIV-1 gene expression and replication by interfering with cyclin-dependent kinase 9 (CDK9)-cyclin T1 interaction in Nef-dependent manner. *J. Biol. Chem.* **2011,** *286*, 22943-22954.
28. Zhang, J.; Li, G.; Ye, X., Cyclin T1/CDK9 interacts with influenza A virus polymerase and facilitates its association with cellular RNA polymerase II. *J. Virol.* **2010,** *84*, 12619-12627.
29. Klebl, B. M.; Choidas, A., CDK9/cyclin T1: a host cell target for antiretroviral therapy. **2006**.

30. Kindrachuk, J.; Ork, B.; Hart, B. J.; Mazur, S.; Holbrook, M. R.; Frieman, M. B.; Traynor, D.; Johnson, R. F.; Dyall, J.; Kuhn, J. H., Antiviral potential of ERK/MAPK and PI3K/AKT/mTOR signaling modulation for Middle East respiratory syndrome coronavirus infection as identified by temporal kinome analysis. *Antimicrob. Agents Chemother.* **2015,** *59*, 1088-1099.
31. Varshney, B.; Lal, S. K., SARS-CoV accessory protein 3b induces AP-1 transcriptional activity through activation of JNK and ERK pathways. *Biochemistry* **2011,** *50*, 5419-5425.
32. Johnson, T. R.; McLellan, J. S.; Graham, B. S., Respiratory syncytial virus glycoprotein G interacts with DC-SIGN and L-SIGN to activate ERK1 and ERK2. *J. Virol.* **2012,** *86*, 1339-1347.
33. Chen, W.; Monick, M. M.; Carter, A. B.; Hunninghake, G. W., Activation of ERK2 by respiratory syncytial virus in A549 cells is linked to the production of interleukin 8. *Exp. Lung Res.* **2000,** *26*, 13-26.
34. Martin, S.; Harris, D. T.; Shisler, J., The C11R gene, which encodes the vaccinia virus growth factor, is partially responsible for MVA-induced NF-κB and ERK2 activation. *J. Virol.* **2012,** *86*, 9629-9639.
35. Takahashi, Y.; Hayakawa, A.; Sano, R.; Fukuda, H.; Harada, M.; Kubo, R.; Okawa, T.; Kominato, Y., Histone deacetylase inhibitors suppress ACE2 and ABO simultaneously, suggesting a preventive potential against COVID-19. *Sci. Rep.* **2021,** *11*, 1-9.
36. Murthy, P. K.; Sivashanmugam, K.; Kandasamy, M.; Subbiah, R.; Ravikumar, V., Repurposing of histone deacetylase inhibitors: A promising strategy to combat pulmonary fibrosis promoted by TGF-β signalling in COVID-19 survivors. *Life Sci.* **2020**, 118883.
37. Sivashanmugam, K.; Kandasamy, M.; Subbiah, R.; Ravikumar, V., Repurposing of histone deacetylase inhibitors: A promising strategy to combat pulmonary fibrosis promoted by TGF-ß signalling in COVID-19 survivors. *Life Sci* **2021**, 118883-118883.
38. Mohamed, M. F.; Shaykoon, M. S. A.; Abdelrahman, M. H.; Elsadek, B. E.; Aboraia, A. S.; Abuo-Rahma, G. E.-D. A., Design, synthesis, docking studies and biological evaluation of novel chalcone derivatives as potential histone deacetylase inhibitors. *Bioorganic Chem.* **2017,** *72*, 32-41.
39. Orlikova, B.; Schnekenburger, M.; Zloh, M.; Golais, F.; Diederich, M.; Tasdemir, D., Natural chalcones as dual inhibitors of HDACs and NF-κB. *Oncol. Rep.* **2012,** *28*, 797-805.
40. Coelho, A. R.; Oliveira, P. J. *Dihydroorotate Dehydrogenase Inhibitors in SARS-CoV-2 Infection*; Wiley: Hoboken, NJ, USA, 2020.
41. Berber, B.; Doluca, O., A comprehensive drug repurposing study for COVID19 treatment: novel putative dihydroorotate dehydrogenase inhibitors show association to serotonin–dopamine receptors. *Brief. Bioinform.* **2021**.
42. Luban, J.; Sattler, R. A.; Mühlberger, E.; Graci, J. D.; Cao, L.; Weetall, M.; Trotta, C.; Colacino, J. M.; Bavari, S.; Strambio-De-Castillia, C., The DHODH inhibitor PTC299 arrests SARS-CoV-2 replication and suppresses induction of inflammatory cytokines. *Virus Res.* **2021,** *292*, 198246.
43. Xiong, R.; Zhang, L.; Li, S.; Sun, Y.; Ding, M.; Wang, Y.; Zhao, Y.; Wu, Y.; Shang, W.; Jiang, X., Novel and potent inhibitors targeting DHODH are broad-spectrum antivirals against RNA viruses including newly-emerged coronavirus SARS-CoV-2. *Protein Cell* **2020,** *11*, 723-739.
44. Xiong, R.; Zhang, L.; Li, S.; Sun, Y.; Ding, M.; Wang, Y.; Zhao, Y.; Wu, Y.; Shang, W.; Jiang, X., Novel and potent inhibitors targeting DHODH, a rate-limiting enzyme in de novo pyrimidine biosynthesis, are broad-spectrum antiviral against RNA viruses including newly emerged coronavirus SARS-CoV-2. *BioRxiv* **2020**.

45. Hahn, F.; Wangen, C.; Häge, S.; Peter, A. S.; Dobler, G.; Hurst, B.; Julander, J.; Fuchs, J.; Ruzsics, Z.; Überla, K., IMU-838, a Developmental DHODH Inhibitor in Phase II for Autoimmune Disease, Shows Anti-SARS-CoV-2 and Broad-Spectrum Antiviral Efficacy In Vitro. *Viruses* **2020,** *12*, 1394.
46. Calistri, A.; Luganini, A.; Conciatori, V.; Del Vecchio, C.; Sainas, S.; Boschi, D.; Lolli, M. L.; Gribaudo, G.; Parolin, C., The new generation hDHODH inhibitor MEDS433 hinders the in vitro replication of SARS-CoV-2. *bioRxiv* **2020**.
47. Xia, C.; Wolf, J. J.; Vijayan, M.; Studstill, C. J.; Ma, W.; Hahm, B., Casein kinase 1$\alpha$ mediates the degradation of receptors for type I and type II interferons caused by hemagglutinin of influenza A virus. *J. Virol.* **2018,** *92*.
48. Quintavalle, M.; Sambucini, S.; Summa, V.; Orsatti, L.; Talamo, F.; De Francesco, R.; Neddermann, P., Hepatitis C virus NS5A is a direct substrate of casein kinase I-$\alpha$, a cellular kinase identified by inhibitor affinity chromatography using specific NS5A hyperphosphorylation inhibitors. *J. Biol. Chem.* **2007,** *282*, 5536-5544.
49. Bhattacharya, D.; Ansari, I. H.; Striker, R., The flaviviral methyltransferase is a substrate of Casein Kinase 1. *Virus Res.* **2009,** *141*, 101-104.
50. Bhargavan, B.; Kanmogne, G. D., Epigenetics, N-myrystoyltransferase-1 and casein kinase-2-alpha modulates the increased replication of HIV-1 CRF02_AG, compared to subtype-B viruses. *Sci. Rep.* **2019,** *9*, 1-19.
51. Zhang, L.; Li, H.; Chen, Y.; Gao, X.; Lu, Z.; Gao, L.; Wang, Y.; Gao, Y.; Gao, H.; Liu, C., The down-regulation of casein kinase 1 alpha as a host defense response against infectious bursal disease virus infection. *Virology* **2017,** *512*, 211-221.
52. Campagna, M.; Budini, M.; Arnoldi, F.; Desselberger, U.; Allende, J. E.; Burrone, O. R., Impaired hyperphosphorylation of rotavirus NSP5 in cells depleted of casein kinase 1$\alpha$ is associated with the formation of viroplasms with altered morphology and a moderate decrease in virus replication. *J. Gen. Virol.* **2007,** *88*, 2800-2810.
53. Chlamydas, S.; Papavassiliou, A. G.; Piperi, C., Epigenetic mechanisms regulating COVID-19 infection. *Epigenetics* **2021,** *16*, 263-270.
54. Tian, R.; Samelson, A. J.; Rezelj, V. V.; Chen, M.; Ramadoss, G. N.; Guo, X.; Mac Kain, A.; Tran, Q. D.; Lim, S. A.; Lui, I., BRD2 inhibition blocks SARS-CoV-2 infection in vitro by reducing transcription of the host cell receptor ACE2. *bioRxiv* **2021**.
55. O'Meara, M. J.; Guo, J. Z.; Swaney, D. L.; Tummino, T. A.; Hüttenhain, R., A SARS-CoV-2-human protein-protein interaction map reveals drug targets and potential drug-repurposing. *BioRxiv* **2020**.
56. Acharya, A.; Pandey, K.; Thurman, M.; Challagundala, K. B.; Vann, K. R.; Kutateladze, T. G.; Morales, G. A.; Durden, D. L.; Byrareddy, S. N., Highly potent PI3K-$\alpha$/mTOR/BRD4 inhibitor for the targeted inhibition of SARS-CoV-2. *bioRxiv* **2021**.
57. Acharya, A.; Pandey, K.; Thurman, M.; Challagundla, K. B.; Vann, K. R.; Kutateladze, T. G.; Morales, G. A.; Durden, D. L.; Byrareddy, S. N., Blockade of SARS-CoV-2 infection in-vitro by highly potent PI3K-$\alpha$/mTOR/BRD4 inhibitor. *bioRxiv* **2021**.
58. Hashimoto, K., Repurposing of CNS drugs to treat COVID-19 infection: targeting the sigma-1 receptor. *Eur. Arch. Psychiatry Clin. Neurosci.* **2021**, 1–10.
59. Vela, J. M., Repurposing sigma-1 receptor ligands for Covid-19 therapy? *Front. Pharmacol.* **2020,** *11*, 1716.
60. Abate, C.; Niso, M.; Abatematteo, F. S.; Contino, M.; Colabufo, N. A.; Berardi, F., PB28, the Sigma-1 and Sigma-2 Receptors Modulator With Potent Anti–SARS-CoV-2 Activity: A Review About Its Pharmacological Properties and Structure Affinity Relationships. *Front. Pharmacol.* **2020,** *11*.

61. Yesilkaya, U. H.; Balcioglu, Y. H.; Sahin, S., Reissuing the sigma receptors for SARS-CoV-2. *J. Clin. Neurosci.* **2020,** *80*, 72-73.
62. Salerno, J. A.; Torquato, T.; Temerozo, J. R.; Goto-Silva, L.; Mendes, M.; Sacramento, C. Q.; Fintelman-Rodrigues, N.; Vitoria, G.; Souza, L.; Ornelas, I., Inhibition of SARS-CoV-2 infection in human cardiomyocytes by targeting the Sigma-1 receptor disrupts cytoskeleton architecture and contractility. *bioRxiv* **2021**.
63. Hashimoto, K., Review 2:" Inhibition of SARS-CoV-2 infection in human cardiomyocytes by targeting the Sigma-1 receptor disrupts cytoskeleton architecture and contractility". *Rapid Rev. COVID-19* **2021**.
64. Gordon, D. E.; Jang, G. M.; Bouhaddou, M.; Xu, J.; Obernier, K.; White, K. M.; O'Meara, M. J.; Rezelj, V. V.; Guo, J. Z.; Swaney, D. L., A SARS-CoV-2 protein interaction map reveals targets for drug repurposing. *Nature* **2020,** *583*, 459-468.
65. Su, H.; Yao, S.; Zhao, W.; Li, M.; Liu, J.; Shang, W.; Xie, H.; Ke, C.; Gao, M.; Yu, K., Discovery of baicalin and baicalein as novel, natural product inhibitors of SARS-CoV-2 3CL protease in vitro. *bioRxiv* **2020**.
66. Abdallah, H. M.; El-Halawany, A. M.; Sirwi, A.; El-Araby, A. M.; Mohamed, G. A.; Ibrahim, S. R.; Koshak, A. E.; Asfour, H. Z.; Awan, Z. A.; Elfaky, M. A., Repurposing of Some Natural Product Isolates as SARS-COV-2 Main Protease Inhibitors via In Vitro Cell Free and Cell-Based Antiviral Assessments and Molecular Modeling Approaches. *Pharmaceuticals* **2021,** *14*, 213.
67. Ullrich, S.; Nitsche, C., The SARS-CoV-2 main protease as drug target. *Bioorganic Med. Chem. Lett.* **2020**, 127377.
68. ul Qamar, M. T.; Alqahtani, S. M.; Alamri, M. A.; Chen, L.-L., Structural basis of SARS-CoV-2 3CLpro and anti-COVID-19 drug discovery from medicinal plants. *J. Pharm. Anal.* **2020,** *10*, 313-319.
69. Shin, D.; Mukherjee, R.; Grewe, D.; Bojkova, D.; Baek, K.; Bhattacharya, A.; Schulz, L.; Widera, M.; Mehdipour, A. R.; Tascher, G., Papain-like protease regulates SARS-CoV-2 viral spread and innate immunity. *Nature* **2020,** *587*, 657-662.
70. McClain, C. B.; Vabret, N., SARS-CoV-2: the many pros of targeting PLpro. *Signal Transduct. Target. Ther.* **2020,** *5*, 1-2.
71. Klemm, T.; Ebert, G.; Calleja, D. J.; Allison, C. C.; Richardson, L. W.; Bernardini, J. P.; Lu, B. G.; Kuchel, N. W.; Grohmann, C.; Shibata, Y., Mechanism and inhibition of the papain-like protease, PLpro, of SARS-CoV-2. *EMBO J.* **2020,** *39*, e106275.
72. Rut, W.; Lv, Z.; Zmudzinski, M.; Patchett, S.; Nayak, D.; Snipas, S. J.; El Oualid, F.; Huang, T. T.; Bekes, M.; Drag, M., Activity profiling and crystal structures of inhibitor-bound SARS-CoV-2 papain-like protease: A framework for anti–COVID-19 drug design. *Sci. Adv.* **2020,** *6*, eabd4596.


**Disclaimer/Publisher's Note:** The statements, opinions and data contained in all publications are solely those of the individual author(s) and contributor(s) and not of MDPI and/or the editor(s). MDPI and/or the editor(s) disclaim responsibility for any injury to people or property resulting from any ideas, methods, instructions or products referred to in the content.
